# Differential plant invasiveness is not always driven by host promiscuity with bacterial symbionts

**DOI:** 10.1093/aobpla/plw060

**Published:** 2016-08-17

**Authors:** Metha M. Klock, Luke G. Barrett, Peter H. Thrall, Kyle E. Harms

**Affiliations:** 1 Department of Biological Sciences, Louisiana State University, Baton Rouge, LA 70803, USA; 2 CSIRO Agriculture Flagship, Canberra, ACT 2601, Australia

**Keywords:** Acacia, biological invasions, interactions, invasive, legume, mutualisms, rhizobia

## Abstract

Identification of mechanisms that allow some species to outcompete others is a fundamental goal in ecology and invasive species management. One useful approach is to examine congeners varying in invasiveness in a comparative framework across native and invaded ranges. *Acacia* species have been widely introduced outside their native range of Australia, and a subset of these species have become invasive in multiple parts of the world. Within specific regions, the invasive status of these species varies. Our study examined whether a key mechanism in the life history of *Acacia* species, the legume-rhizobia symbiosis, influences acacia invasiveness on a regional scale. To assess the extent to which species varying in invasiveness correspondingly differ with regard to the diversity of rhizobia they associate with, we grew seven *Acacia* species ranging in invasiveness in California in multiple soils from both their native (Australia) and introduced (California) ranges. In particular, the aim was to determine whether more invasive species formed symbioses with a wider diversity of rhizobial strains (i.e. are more promiscuous hosts). We measured and compared plant performance, including aboveground biomass, survival, and nodulation response, as well as rhizobial community composition and richness. Host promiscuity did not differ among invasiveness categories. *Acacia* species that varied in invasiveness differed in aboveground biomass for only one soil and did not differ in survival or nodulation within individual soils. In addition, acacias did not differ in rhizobial richness among invasiveness categories. However, nodulation differed between regions and was generally higher in the native than introduced range. Our results suggest that all *Acacia* species introduced to California are promiscuous hosts and that host promiscuity *per se* does not explain the observed differences in invasiveness within this region. Our study also highlights the utility of assessing potential mechanisms of invasion in species’ native and introduced ranges.

## Abstract (precis)

Acacias have been widely introduced outside their native range, with a subset of species becoming invasive in multiple parts of the world. Our study examined whether a key mechanism in acacia life history, the legume-rhizobia symbiosis, influences invasiveness of these species. We determined whether more invasive acacias formed symbioses with a wider diversity of rhizobial strains (i.e. are more promiscuous hosts) and found that acacias introduced to California are promiscuous hosts regardless of invasive status. Our results highlight the importance of examining mechanisms driving species invasions on different scales and in their native and introduced ranges.

## Introduction

Non-native species are a threat to native ecosystems, particularly when they colonize new areas and rapidly expand in abundance. Collectively, invasive species have negative impacts at both local and global scales, threatening biodiversity, accelerating global change and causing economic losses (D'Antonio and Vitousek 1992; Vitousek *et al.* 1996; Mack *et al.* 2000; Pimentel *et al.* 2000). Although not all introduced species become invasive, those that do variously alter food sources for native wildlife, change fire regimes, outcompete native species, and impact soil communities, for example, by altering microbial structure and soil nitrogen levels (Mack and D'Antonio 1998; Mack *et al.* 2000; Brooks *et al.* 2004). To better understand how species become invasive in new environments, in-depth investigations of mechanisms driving species invasions are needed.

Diverse mechanisms and hypotheses have been proposed for why introduced species become invasive. Many of the better-investigated drivers of invasiveness are based on antagonistic or competitive interactions (Blossey and Notzold 1995; Callaway and Aschehoug 2000; Keane and Crawley 2002; Levine *et al.* 2003). Much work to date has investigated the role of enemy-release in facilitating species invasions (i.e. invaders that prosper in new environments because they leave their parasites, pests, and predators behind [Keane and Crawley 2002]). The Evolution of Increased Competitive Ability Hypothesis predicts that adaptive evolution of invaders provides a competitive advantage in novel ranges (Blossey and Notzold 1995). Although overcoming adversity imposed by antagonists and competitors may be the driver of invasiveness for some species, mutualistic interactions may also play a key alternate or synergistic role in some invasions (Richardson *et al.* 2000).

A growing body of work has examined the role of mutualisms in the invasion of non-native species (Richardson *et al.* 2000; Birnbaum *et al.* 2012; Wandrag 2012). The Enhanced Mutualism Hypothesis proposes that species encounter novel beneficial symbionts in their native range, which enhance their ability to survive and spread abroad (Richardson *et al.* 2000). The Accompanying Mutualist Hypothesis suggests that invasive species are introduced concurrently with their native mutualistic partners, thereby enhancing their ability to survive in novel habitats (Rodríguez-Echeverría 2010). Mutualisms such as those between legumes and their symbiotic nitrogen-fixing soil bacteria (i.e. rhizobia) may be particularly important in explaining the ability of this group of species to establish and expand abroad. Elucidating the potential role that mutualistic interactions play in species establishment and colonization may point towards mechanisms driving differential levels of species invasion.

Australian *Acacia* species (Family: Fabaceae) are a diverse group of legumes that form symbiotic relationships with rhizobia. They have been introduced throughout the world for a variety of purposes, including ornamental use, fuel wood, erosion control, and forestry (Kull and Rangan 2008; Carruthers *et al.* 2011). Many *Acacia* species that have been introduced outside their native range have become invasive abroad (Richardson *et al.* 2011). Of the more than 1000 *Acacia* species occurring in Australia (Miller *et al.* 2011), ∼400 species have been introduced outside their native range, with ∼6 % becoming invasive, ∼12 % becoming naturalized and ∼82 % remaining as casuals (Richardson *et al.* 2011; Rejmánek and Richardson 2013) (see Table 1 for definition of invasiveness categories).

**Table 1.** Definition of the terms “invasive,” “naturalized” and “casual” as they relate to the invasiveness categories of *Acacia* species introduced to novel ranges.

Term	Definition	Reference
Invasive	Non-native species that (1) have self-sustaining populations which, for a minimum of 10 years have reproduced by seed or ramets without (or despite) human intervention, and (2) have spread and established reproductive populations at large distances from parent plants	Richardson *et al*. (2011)
Naturalized	Non-native species that have escaped cultivation and established self-sustaining populations but have not spread to the extent of invasive species	Richardson *et al*. (2011)
Casual	Non-native species that do not establish populations without the aid of humans (also ‘waifs’)	Richardson *et al*. (2000); Jepson Flora Project (2015)

Globally, acacias vary in the number of regions they have invaded [regions defined by Richardson and Rejmánek (2011) and Rejmánek and Richardson (2013) include North America, Europe, Middle East, Asia, Indonesia, Pacific Islands, New Zealand, Australia, Indian Ocean Islands, Africa (southern), Africa (rest), Atlantic Islands, South America, Caribbean Islands, and Central America]. Differences in acacia invasiveness among regions may be due to variation in invasive capacity of these species, lower propagule pressure in particular regions or differences in incidence reports among regions (Richardson and Rejmánek 2011).

Within geographic regions, there is also evidence that acacias vary in invasiveness. For example, sixteen Australian *Acacia* species have been introduced to California and differ in their invasive status in this region (Jepson Flora Project 2015) (Table 2). Whereas all these species except for two (*A. cultriformis* and *A. redolens*) are invasive in at least one part of the world, they vary markedly in their ability to invade and expand population sizes in California. *Acacia* species were first introduced to California for ornamental purposes and sold through the nursery trade beginning in the mid-1800s (Butterfield 1938). Two species, *A. dealbata* and *A. melanoxylon*, are currently designated as invasive in California (Cal-IPC 2006), five species as naturalized and nine species as casuals (Jepson Flora Project 2015) (Table 2). Definitions of invasiveness categories used for the purpose of this study can be found in Table 1. Understanding the mechanisms that enable multiple closely related species to differentially establish and colonize natural areas in one particular region is important for understanding what controls and promotes species establishment in general (Klock *et al.* 2015).

**Table 2.** *Acacia* species occurring in California. California invasiveness status compiled from CalFlora (CalFlora 2015), Cal-IPC (Cal-IPC 2006) and Jepson herbarium (Jepson Flora Project 2015). Regions invaded globally compiled from Richardson *et al*. (2011) and Rejmanek *et al*. (2013) [Regions include: North America, Europe, Middle East, Asia, Indonesia, Pacific Islands, New Zealand, Indian Ocean Islands (including Madagascar), Africa (southern), Africa (rest), Atlantic islands, South America, Caribbean islands, and Central America]. *Acacia* species included in this study are noted with an *.

Species	California status	Regions invaded globally
A. baileyana*	Naturalized	2
A. cultriformis*	Casual	0
*A. cyclops*	Naturalized	4
A. dealbata*	Invasive	6
*A. decurrens*	Casual	3
*A. elata*	Casual	1
A. longifolia*	Naturalized	7
*A. mearnsii*	Casual	12
A. melanoxylon*	Invasive	10
*A. paradoxa*	Casual	4
*A. podalyriifolia*	Casual	2
A. pycnantha*	Casual	2
*A. redolens*	Naturalized	0
*A. retinodes*	Casual	2
*A. saligna*	Naturalized	4
A. verticillata*	Casual	2

One mechanism that may be an important determinant of invasion success for acacias is their symbiotic relationship with rhizobia. The legume–rhizobia interaction has been long recognized as critical for the growth and establishment of many legumes (Sprent 2001), including acacias (Thrall *et al.* 2005). Rhizobia are Gram-negative bacteria that convert atmospheric nitrogen to a form usable by the plant (Bauer 1981; Sprent and Sprent 1990). Within nodules, the plant provides rhizobia access to carbon substrates and micronutrients, and also protects them from desiccation (Sprent 2001). When legumes form an association with compatible symbiotic bacteria they obtain a direct source of nitrogen unavailable to other plants. Soil nitrogen availability for plants is often low (Masclaux-Daubresse *et al.* 2010), so species that are more readily able to form such associations may have a competitive advantage over other plant species, particularly in low-resource environments (Funk and Vitousek 2007).

The selectivity of different plant hosts for particular rhizobial symbionts (hereafter referred to as “host promiscuity”) may contribute to the differential ability of *Acacia* species to establish and expand abroad. Hosts that are more promiscuous (i.e. are able to effectively associate with a wider range of rhizobial strains) may have a competitive advantage when introduced to novel areas, where they are likely to encounter unfamiliar nitrogen-fixing bacteria (Richardson *et al.* 2000; Rodríguez-Echeverría 2010; Birnbaum *et al.* 2012). Previous research suggests that widely distributed acacias in their native range are more promiscuous rhizobial hosts, whereas those with more limited distribution are more specific hosts (Thrall *et al.* 2000). In addition, acacias that have become invasive in multiple regions of the globe appear to be more promiscuous hosts than naturalized or casual acacias (Klock *et al.* 2015). Variation in host promiscuity among *Acacia* species introduced to California may help explain why certain species have differentially invaded this region.

The goal of this study was to characterize the nodulation ability of a suite of *Acacia* species that have become differentially invasive within California. To examine this, we used multiple *Acacia* species representing different invasiveness categories and performed whole soil inoculation experiments with a range of soils from different environments and two different continents. Examining species in their native and introduced ranges can provide essential information for understanding the context-dependent mechanisms influencing the invasion of non-native species (Shea *et al.* 2005). By better understanding the biological attributes of species in their home range, we can predict and compare their responses abroad, thereby gaining insight into which mechanisms are influencing species survival and expansion in different ranges (Hierro *et al.* 2005). Using species of *Acacia* and their rhizobial mutualists, we aimed to assess whether the mechanisms promoting establishment and survival at home are the same that facilitate invasion abroad. The purpose of conducting this experiment in the native range was to challenge acacias with unfamiliar rhizobial communities in areas where they naturally occur. This mimics the conditions legume hosts face when introduced abroad (although potential rhizobial mutualists are likely to be more closely related to those they typically associate with). Our approach also allowed us to determine if observed patterns are maintained in the invasive range, where rhizobial mutualists may be more distantly related.

In particular, we evaluated aboveground plant growth (biomass), survival, and nodulation responses. We examined whether treatment of acacias with different soil inoculants influenced plant performance. We also used terminal restriction length polymorphism (T-RFLP), to examine the composition and richness of rhizobial strains associating with acacias in different invasiveness categories. We hypothesized that invasiveness of non-native acacias in California would be influenced by host promiscuity with rhizobial strains, with the following predictions: (1) invasive acacias would have higher biomass, survival and nodulation responses (i.e. plant performance) in both native and introduced ranges across a greater number of soils than naturalized or casual acacias; and (2) invasive acacias would associate with a greater number of rhizobial strains (as measured by number of ribotypes, or unique terminal restriction fragment lengths) in both native and introduced ranges than naturalized or casual species.

## Methods

### Study species

**Figure 1.**
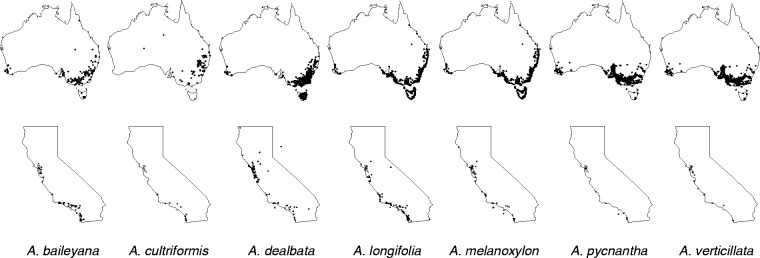
Distribution maps for *Acacia* species used in this experiment in their native continent of Australia (top row) (based on herbarium records from the Australian National Herbarium, Canberra, Australia [AVH 2015]) and introduced range of California (bottom row) (Data provided by the participants of the Consortium of California Herbaria [ucjeps.berkeley.edu/consortium/, last accessed 04 August 2016.]).

The genus *Acacia* (Fabaceae: Mimosoideae) is native to Australia, with over 1000 species occurring variously across the continent (Miller *et al.* 2011) (Fig. 1). We focused on seven species that have been introduced to California and have become invasive (*A. dealbata* and *A. melanoxylon*), naturalized (*A. baileyana* and *A. longifolia*) or remained casual aliens (*A. cultriformis*, *A. pycnantha* and *A. verticillata*) in this region (Cal-IPC 2006; Jepson Flora Project 2015) (see Fig. 1 for *Acacia* range distributions in Australia and California). Five of these species have been previously characterized for levels of host promiscuity (*A. dealbata*, *A. cultriformis*, *A. longifolia*, *A. melanoxylon* and *A. pycnantha*) using pure rhizobial cultures (Thrall *et al.* 2000; Bever *et al.* 2013; Klock *et al.* 2015), whereas two species have not (*A. baileyana* and *A. verticillata*). All species examined here are native to southeastern Australia and range from broadly distributed to narrowly restricted within their native region (AVH 2015) (Fig. 1). Previous research has provided at least some evidence that more widely distributed acacias in their native range are more promiscuous rhizobial hosts than those that are narrowly distributed (Thrall *et al.* 2000), and that globally invasive acacias are more promiscuous hosts than those that are naturalized or casual aliens (Klock et al. 2015). Given the analogous variation in the occurrence of our selected species within their novel range, we used these species to examine whether invasiveness in California might also be linked to variation in host promiscuity.

### Soil inoculant collection and preparation

**Figure 2.**
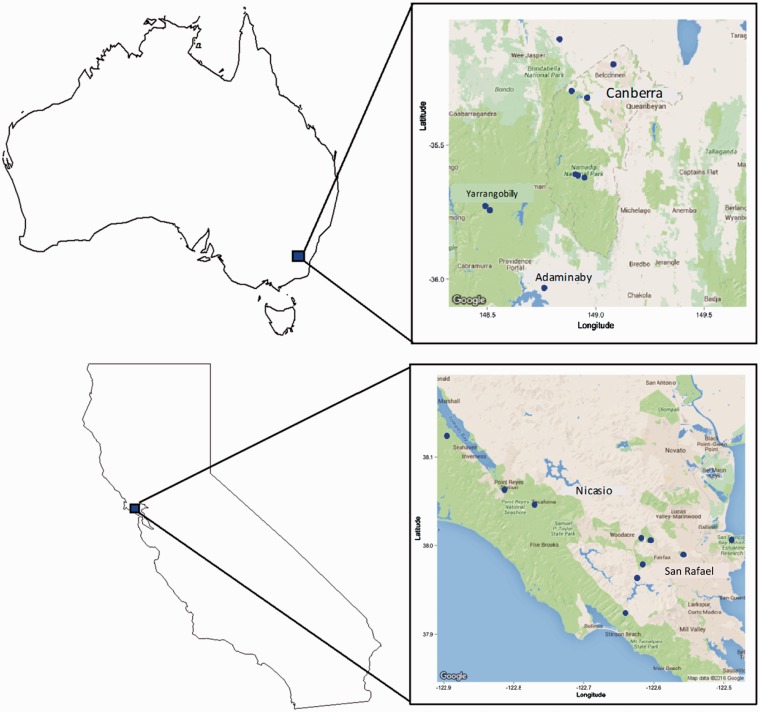
Soil inoculation collection sites in Australia (top) and California (bottom). Maps created using the R statistical package “ggmap” version 2.5.2 (Kahle and Wickham 2013).

Soil samples were collected from multiple sites in *Acacia* species’ native (Australia) and introduced (California) ranges to obtain a diverse suite of rhizobial communities for use in glasshouse inoculation studies (Fig. 2 **[see Supporting Information—Table S1]**). Whole soil inoculations were used rather than individual rhizobial cultures to challenge acacias with rhizobial communities they have not previously been exposed to, thereby reflecting more accurately the conditions acacias may face when introduced abroad. Soils likely contained organisms other than just rhizobia; however, all soils were bulked and mixed within soil collection site, and all *Acacia* species inoculated with soils from each site to achieve homogenous treatment conditions.

In Australia, we collected soils from ten sites within a 150 km radius of Canberra, ACT, during July 2011. Sites varied in disturbance regimes, from a highly disturbed agricultural field, to an abandoned paddock, to an undisturbed diverse native legume site. In California, we collected soils from ten sites within a 50 km radius of San Francisco, CA, during December 2011 (Fig. 2; **[see Supporting Information—Table S1]**). Weather conditions in Australia and California were very similar during the sampling periods (high temp 11.2 °C vs. 14 °C; low temp −1.4 °C vs. 1.7 °C; precipitation 0.04 cm vs. nil) (www.ncdc.noaa.gov, last accessed 03 August 2016).

In both ranges, we chose sites that did not contain any of the *Acacia* species used in this study to challenge all of the study species with unfamiliar rhizobial communities. This was done to mimic conditions that hosts might encounter when introduced to a new area. Soils were collected over the course of one week. Soil samples were excavated using a clean shovel and stored in paper bags until processing. We collected multiple samples from within each site and then bulked them within replicates, with site as the level of replication, to make a single composite for each of the 10 sites. Following collection, soils were dried for up to 6 days. Once dry, they were sieved through 3-mm mesh to remove rocks and other debris and stored in paper bags until use. Soils collected in California were shipped to Louisiana State University (LSU) for use in the introduced range glasshouse experiment. Temperatures at which soils were stored fluctuated due to transport and handling requirements but otherwise were held constant at 4°C. Previous research has shown that the abundance of rhizobial strains can decline over time in dry soil storage; however, rhizobial strains are still abundant in soils after 6 months (Martyniuk and Oroń 2008; Thrall and Barrett pers. obs.). In addition, as each *Acacia* species was subject to each soil treatment, exposure to available rhizobial strains was the same among species.

### Glasshouse experiments

We conducted two glasshouse experiments to examine the promiscuity of *Acacia* species in different invasiveness categories. For the first experiment (hereafter called the “native experiment”), glasshouse facilities were located at CSIRO’s Black Mountain site in Canberra, ACT, Australia. For the second experiment (hereafter called the “introduced experiment”), glasshouse facilities were located at LSU in Baton Rouge, Louisiana. The native experiment was conducted from July to November 2011. Seeds of all *Acacia* species used in this experiment were collected within Australia and obtained from the Australian Seed Company. The introduced experiment was conducted from March to July 2012. For this component, seeds of all *Acacia* species were collected directly from plants in California in September 2011, shipped to LSU, and stored in paper bags until use.

For both experiments, seeds were subjected to a boiling water treatment to induce germination (boiling water was poured over seeds and they were left to imbibe water for 24 h). No further seed sterilization methods were undertaken; however, seedlings were observed for nodule presence at time of planting and none were nodulated. In addition, while the native experiment control did experience a moderate level of contamination at final harvest, samples in the introduced range control treatment showed no contamination, suggesting that the source of contamination for the native experiment was not the vertical transmission of rhizobia. Seeds were transferred to trays of steam-sterilized vermiculite and watered daily with sterile water for 14–20 days, or until germination occurred. Seedlings were grown in the glasshouse under local natural light conditions.

Once germinated, seedlings were transferred to individual pots inoculated with soils collected from each of the 10 sites. In the native experiment, for each of the bulked soils, 10 replicates of each *Acacia* species were planted in 8 × 15 cm pots filled ¾ with sterilized sand and vermiculite (1:1 volume), 50 g of an individual soil treatment as a live inoculant and topped with additional sterilized sand and vermiculite (1:1 volume) to avoid cross contamination. For the introduced experiment, seedlings were similarly planted and inoculated, however replication varied due to availability of seed for individual species (10 replicates of *A. baileyana, A. longifolia, A. melanoxylon* *and A. verticillata*; 5 replicates of *A. dealbata* and *A. pycnantha*; 4 replicates of *A. cultriformis*). A rhizobia-free (N^–^) control was also included in both experiments in which plants were not inoculated. For both experiments, *Acacia* species × soil combinations were spatially randomized by glasshouse bench such that each bench contained one replicate of each species × soil combination. Pot placement on the bench was randomized. All plants were watered twice weekly with sterile N-free McKnight’s solution (McKnight 1949) and sterile water as needed. Plants were spaced well apart on glasshouse benches to minimize cross-contamination during watering.

Plants were grown for 16 weeks in a temperature-controlled glasshouse (∼20 °C) and harvested in November 2011 (native experiment) and July 2012 (introduced experiment), respectively. At harvest, seedlings were clipped at the soil surface and aboveground material was stored in paper bags. For the native experiment, aboveground material was oven dried at 70 °C for 48 h and weighed. A malfunction with the drying oven destroyed aboveground material for the introduced experiment, therefore biomass data were lost. Belowground material for both experiments (roots and attached nodules) of each plant was stored individually in plastic bags and frozen at –20 °C until processing for molecular analysis. Roots were scored at harvest for nodulation quantity (0, <10, 10–50, >50) and quality (none, ineffective [black or very small white nodules], intermediate [mixture of small to medium white/pink nodules] and good [pink nodules]) (Thrall *et al.* 2011).

### Isolation of DNA and T-RFLP

We used terminal restriction length polymorphism (T-RFLP) to identify community composition and genotypic richness of rhizobia nodulating with *Acacia* species in the glasshouse experiments. This technique is frequently used for examining taxon richness of bacterial communities (Liu *et al.* 1997). To extract DNA from root nodules collected during harvest, 2–10 intact nodules per plant (depending on availability) were first snipped from roots stored at –20 °C. Nodules were surface sterilized by immersion in 90 % ethanol for five to ten seconds, transferred to 3 % sodium hypochlorite and soaked for 2–4 minutes, and rinsed in five changes of sterile water. Nodules were crushed using liquid nitrogen, and DNA was extracted using Mo Bio PowerPlant® DNA Isolation kits following the protocol of the manufacturer (Mo Bio Laboratories, Inc., Carlsbad, CA, USA). Nodule processing and DNA extractions for the native experiment were conducted at CSIRO laboratories in Canberra, Australia, and for the introduced experiment at LSU in Baton Rouge, LA. DNA extractions from the introduced experiment were shipped to CSIRO laboratories where all additional molecular analyses were conducted. For all samples, we amplified the 16S rRNA gene using the primers GM3 (5’-AGA GTT TGA TCM TGG C-3’) and GM4 (5’-TAC CTT GTT ACG ACT T-3’) and the following PCR program: initial denaturation at 95 °C for 2 min, followed by 35 cycles of 95 °C for 30 s, 50 °C for 30 s and 72 °C for 90 s, followed by a final extension step at 72 °C for 10 min and a final holding temperature of 4 °C. We digested the PCR product using the restriction enzyme MspI (New England BioLabs) in 30 μl reaction mixtures, and analysed the fragment sizes using a 3130×l genetic analyzer (Applied Biosystems, Warrington, United Kingdom). We used GeneMapper version 5 (Life Technologies, Grand Island, NY, USA) to examine T-RFLP profiles and included peaks over 50 bp for further analysis. We quantified resulting peaks using the local southern method (Southern 1979). Peaks were binned using Ramette’s interactive binner script (Ramette 2009) in the R statistical programming language version 3.2.0 (R Core Team 2015).

DNA extracted from nodules contained both acacia plastid and rhizobial DNA. While the GM3/GM4 primers can also amplify mitochondrial and chloroplast DNA, in-silico analyses of restriction-fragment polymorphisms for all *Acacia* species plastid sequences obtained from Genbank indicated that polymorphisms in plastid DNA were unlikely to contribute any variation to our T-RFLP dataset. Specifically, to identify which restriction-fragments corresponded to acacia plastid DNA, we conducted an in-silico T-RFLP analysis by searching for the primer sequences and restriction enzyme cut sites in acacia plastid DNA sequences downloaded from GenBank. We found that the restriction enzyme MspI cut sites for acacia plastid sequences generated DNA fragments greater in size than the cut-off for fragments used in our analysis (i.e. the largest restriction-fragment in our analysis was 545.3 bp, whereas the smallest restriction fragment for acacia plastid DNA was 553 bp). Because our cut-off was lower than the largest acacia plastid restriction-fragment, any peaks corresponding to acacia plastid DNA were excluded from our analysis. In addition, review of polymorphisms attributable to individual host species showed there were no polymorphisms unique to all replicates of a host species (or group of host species), further indicating that acacia plastid DNA did not explain variation in the dataset.

### Plant growth, survival and nodulation response

We examined the responses of acacias representing three invasiveness categories to inoculation with 20 different soils (10 soils each in the native and introduced ranges) collected from habitats in which the acacias used in this experiment do not occur. We measured differences among the invasiveness categories by assessing aboveground biomass (native range only), survival, nodulation presence/absence and nodulation index of effectiveness. The nodulation index of effectiveness categorizes the number of nodules found on the roots of plant specimens, and is divided into levels of none, low, medium, and high, delineated as follows: 0 nodules  =  score of 0; 1–10 nodules  =  score of 1; 11–50 nodules  =  score of 2; >50 nodules  =  score of 3.

We examined these four variables for the entire data set using generalized linear mixed models (GLMM) and used AIC to select the best models (Burnham and Anderson 2002). *Acacia* species was included in the models as a random effect to include individual variation of species in each invasiveness category. Aboveground biomass and nodulation index were modelled using a Gaussian distribution, and nodule presence/absence and survival were modelled using a binomial distribution with a logit link function. Negative control samples were not included in models for the native experiment, as almost all control specimens did not survive; however, they were included in models for the introduced experiment.

We used the R statistical package “lme4” version 1.1-9 (Bates *et al.* 2012) to determine whether main effects (soil, invasiveness category and *Acacia* species) contributed significantly to the models of interest, and whether there were interactions among main effects. *Acacia* species was maintained in all models as a random effect. Models with the lowest AIC score were selected for further analysis; models with a difference in AIC values of <2 were considered equally likely (Burnham and Anderson 2002; Bolker *et al.* 2009). Further analysis consisted of conducting multiple comparisons of means (MCMs) with Tukey contrasts using the R statistical package “multcomp” version 1.4-1 (Hothorn *et al.* 2008), which allowed us to determine whether there were significant differences among invasiveness categories for the response variables of interest (i.e*.* biomass, nodulation presence and nodulation index) for individual soils, while maintaining *Acacia* species in the model as a random variable.

We also examined biomass (native experiment only), nodulation presence/absence, and survival for individual *Acacia* species to assess species-specific responses to individual soil inoculants. We used ANOVA to compare biomass among species x soil combinations and logistic regression to analyse survival and nodulation presence. We used a post-hoc Tukey’s HSD test to compare biomass of different species to each soil inoculant using the R statistical package “agricolae” version 1.1-2 (De Mendiburu 2009). Analyses were conducted using the R statistical programming language version 3.2.0 (R Core Team 2015).

### Rhizobial community composition and richness

We analysed binary data obtained from T-RFLP analysis using non-metric multidimensional scaling (NMDS) based on a Jaccard similarity matrix. We used the R statistical package “vegan” version 2.3-0 (Oksanen *et al.* 2015) to conduct ordination and Permutational ANOVA (PerManova; function “ADONIS”) to test for differences in rhizobial community composition among invasiveness categories and soil types. If differences were detected we ran pairwise comparisons between groups using “ADONIS” with a Holm correction. We used ANOVA to examine whether there were differences in ribotype richness among invasiveness categories. Analyses were conducted using the R statistical programming language version 3.2.0 (R Core Team 2015).

## Results

### Native experiment

**Figure 3.**
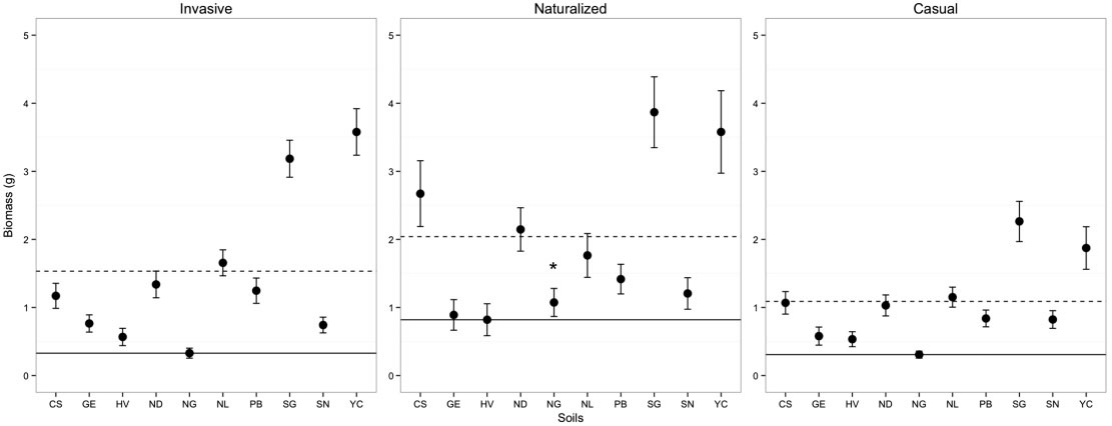
Average aboveground biomass (g) response of all *Acacia* species/replicates in each invasiveness categories to different soil inoculants in the native experiment (Australia). The horizontal solid line indicates the point at which host species within a given invasiveness category have the same biomass response as their least effective soil. The dashed line is the average biomass response for all host species within a given invasiveness category combined across all soils. The “*” indicates the soil in which there was a significant difference (*P* < 0.05) in biomass response of the invasiveness categories. Error bars represent standard errors (SE) of the means.

We detected a significant interaction between soil and invasiveness category for aboveground biomass (ΔAIC  =  19.1, *w_i_*  =  1.00) (i.e. the best fitting model had an AIC value >2 than all other models), indicating that the growth response of species in different invasiveness categories was influenced by the soil in which they were grown **[see Supporting Information—Table S2]**. We, therefore, examined each soil individually using MCMs with Tukey contrasts and found that plants in different invasiveness categories differed significantly in average biomass response for only one soil (Fig. 3 **[see Supporting Information—Table S3]**)

ANOVA results indicated that biomass varied for individual *Acacia* species across soil treatments (*F*_9,605 _ = _ _470.21, *P *< 0.001); we also found a significant difference in biomass across *Acacia* species (*F*_6,608 _ = _ _346.80, *P *< 0.001), and an interaction between species and soil treatment (*F*_54,545 _ = _ _135.01, *P *< 0.001) (Table 3). From here on, individual *Acacia* species are indicated in the text as I (invasive), N (naturalized) and C (casual). Using as a comparison the soil where biomass was lowest for each species, post-hoc Tukey’s HSD test showed that *A. longifolia* (N) and *A. melanoxylon* (I) had significantly greater biomass for three soils, *A. baileyana* (N), *A. cultriformis* (C), *A. dealbata* (I) and *A. verticillata* (C) for two soils, and *A. pycnantha* (C) for one soil **[see Supporting Information—Fig. S1 and Table S4]**.

**Table 3.** Summary of analysis of variance results testing the effects of host species and soil treatment on the aboveground biomass response.

Source	df	SS	*F*	*P*
Host species	69	346.80	61.93	<0.001
Soil	9	470.21	55.98	<0.001
Host x Soil	54	135.01	2.68	<0.001
Residual	545	508.63		

**Figure 4.**
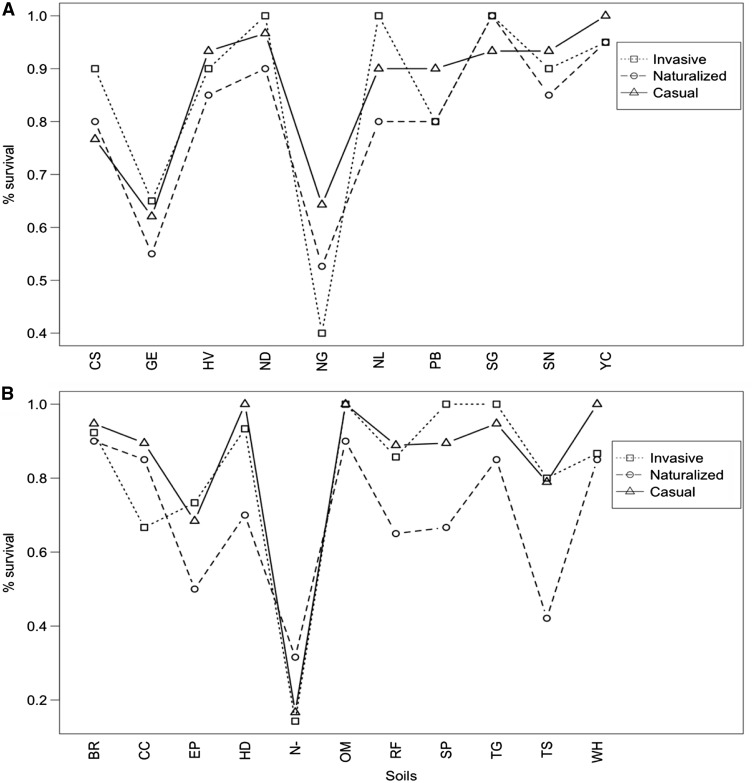
Average percent survival of all *Acacia* species/replicates in each invasiveness category in the (A) native and (B) introduced experiments among soil treatments.

The model with the best support for plant survival included soil inoculation as a main effect with species as a random variable (ΔAIC  =  3.87, *w_i_*  =  0.87) **[see Supporting Information—Table S5A]**, indicating that variation in survival was driven by individual soils rather than invasiveness category. Survival across soils was generally high for all invasiveness categories (>50 % for all soils for the naturalized and casual categories and nine out of ten soils for the invasive category) (Fig. 4A **[see Supporting Information—Table S6A]**).

Survival for individual species was also generally high across soils. We observed over 50 % survival for each species in a minimum of seven soils (*A. pycnantha* [C]) and a maximum of all ten soils (*A. longifolia* [N] and *A. verticillata* [I]) **[see Supporting Information—Fig. S2 and Table S7A]**.

There was a moderate level of contamination in the negative controls (nodules were found on ∼33 % of samples), and very few samples that were not contaminated survived, therefore, they were excluded from all native experiment analyses.

**Figure 5.**
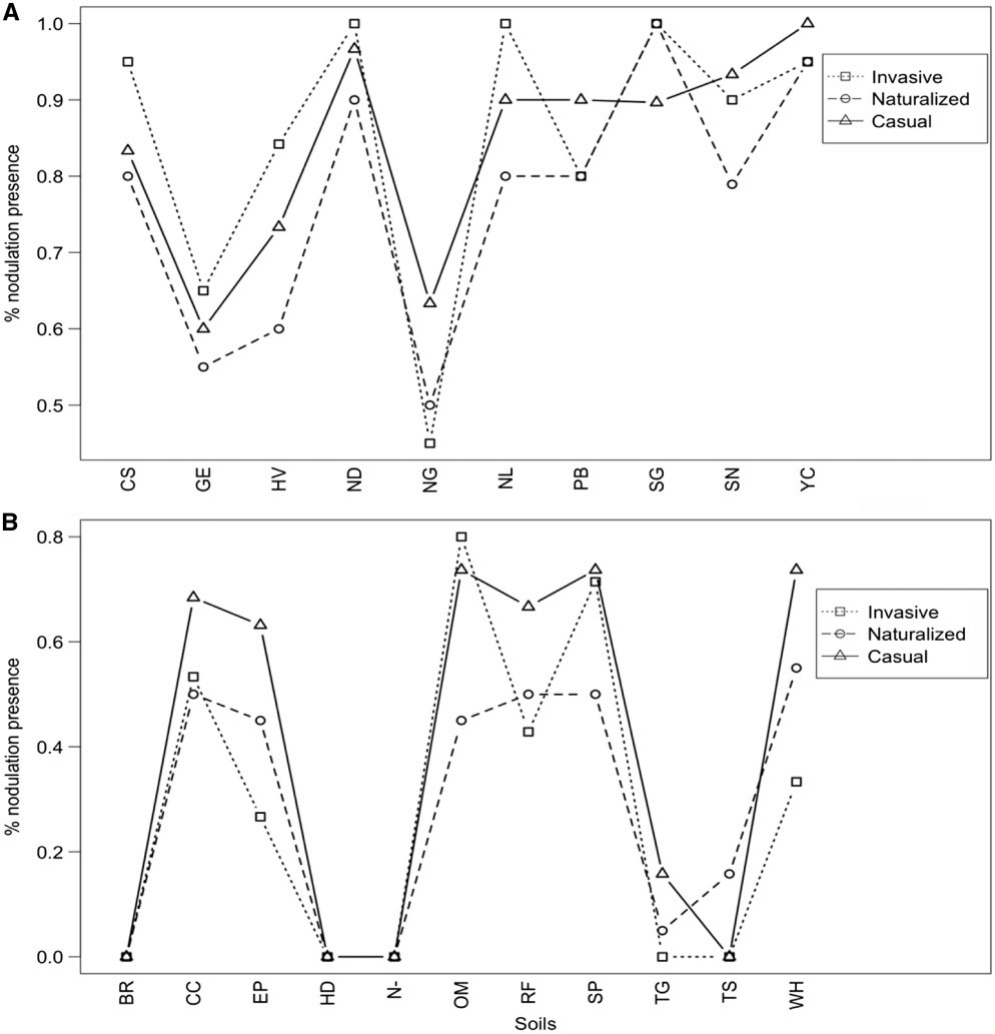
Average percent nodulation of all *Acacia* species/replicates in each invasiveness category in the (A) native and (B) introduced experiments among soil treatments.

The model with best support for nodulation presence included soil inoculation as a main effect with species as a random variable (Native experiment: ΔAIC  =  3.92, *w_i_*  =  0.88) **[see Supporting Information—Table S8A]**, indicating that differences in nodulation presence were driven by individual soils rather than invasiveness category. The presence of nodules across soils was generally high for all invasive categories (>50 % in ten soils for the casual category and nine soils for the naturalized and invasive categories) (Fig. 5A **[see Supporting Information—Table S9A]**).

Nodulation presence for individual species was also generally high across soils, with over 50 % nodulation presence for each species in a minimum of seven soils (*A. baileyana* [N]) and a maximum of all ten soils (*A. cultriformis* [C], A*. longifolia* [N], *A. melanoxylon* [I] and *A. verticillata* [C]) (**see Supporting Information—Fig. S4 and Table S10A]**).

**Figure 6.**
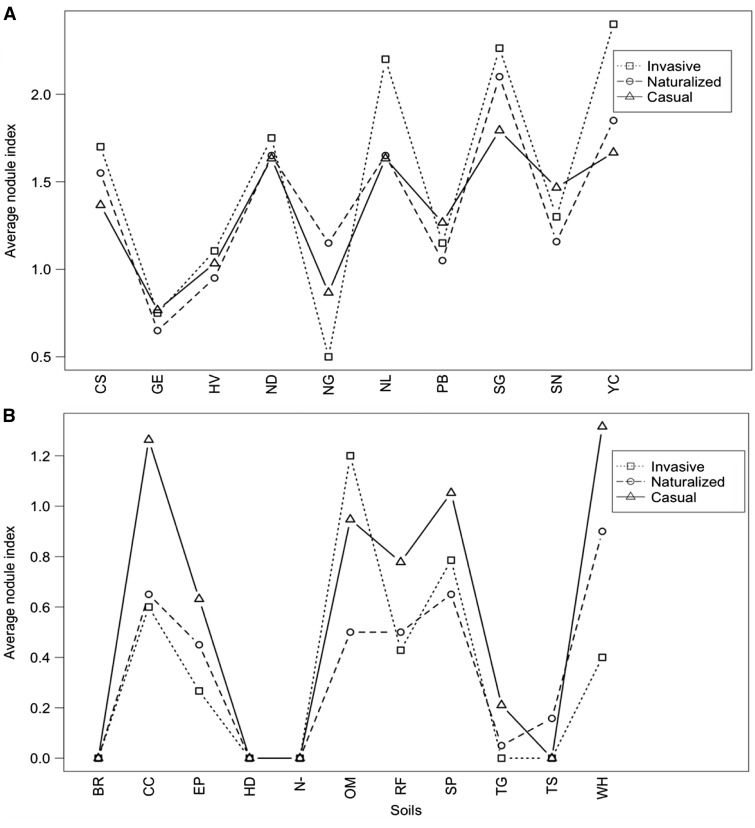
Average nodulation index of all *Acacia* species/replicates in each invasiveness category in the (A) native and (B) introduced experiments among soil treatments. The different shapes depict different invasiveness categories (square = invasive, circle = naturalized, triangle = non-invasive).

We found a significant interaction between soil and invasiveness category for nodulation index of effectiveness (ΔAIC  =  9.7, *w_i_*  =  0.97) **[see Supporting Information—Table S11A]**. This indicates that there was an effect of individual soils on nodulation index, such that the number of nodules on plants belonging to different invasiveness categories depended on the soil in which they were grown. We, therefore, could not generalize nodulation index response for invasiveness categories across all soils, and examined nodulation index for each soil individually using MCMs with Tukey contrasts. When soils were examined individually, we found no significant difference in nodulation index among invasiveness categories (Fig. 6A).

**Figure 7.**
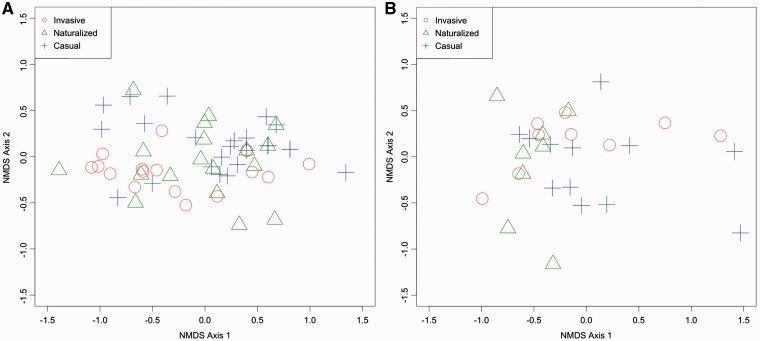
Ordination of the rhizobial community composition in different invasiveness categories (Jaccard similarity) in the (A) native and (B) introduced experiments based on the 16S rRNA gene from different soil treatments derived from nonmetric multidimensional scaling. Invasiveness categories more similar in rhizobial community composition are closer together in ordination space.

Visual assessment of ordination diagrams did not indicate a clear difference in rhizobial community composition among *Acacia* species invasiveness categories (Fig. 7A). However, PerManova results from T-RFLP analyses indicated a small but significant difference in rhizobial community composition between the invasive and casual categories (ADONIS, *R*  =  0.08, adjusted *P*  =  0.024). Despite this slight difference in community composition, there was no significant difference in rhizobial richness among invasiveness categories (*F*  =  1.287, *P*  =  0.284).

### Introduced experiment

No contamination occurred in the introduced range experiment so all control samples were retained for all analyses. The best-supported model for survival in the introduced range experiment included soil inoculation as a main effect with species as a random variable (ΔAIC  =  2.73, *w_i_*  =  0.79) **[see Supporting Information—Table S5B]**. Similar to the native experiment, survival across soils was generally high for all invasiveness categories (>50 % for all soils for the invasive and casual categories, and 8 out of 10 soils for the naturalized category) (Fig. 4B **[see Supporting Information—Table S6B]**).

Survival for individual species was also high across soils. We observed over 50 % survival in a minimum of six soils (*A. baileyana* [N]) and a maximum of all 10 soils (*A. longifolia* [N], *A. melanoxylon* [I] and *A. verticillata* [C]) **[see Supporting Information—Fig. S3 and Table S7B]**.

The model with the best support for nodulation presence included only soil inoculation as a main effect with species as a random variable (ΔAIC  =  3.99, *w_i_*  =  0.88) **[see Supporting Information—Table S8B]**. In contrast to the native experiment, nodule presence across soils was low for the introduced experiment (>50 % in six soils for the casual category, three soils for the invasive category and one soil for the naturalized category) (Fig. 5B **[see Supporting Information—Table S9B]**).

Nodulation presence was generally low across soils for individual species as well, with over 50 % nodulation presence for each species in a maximum of six soils (*A. longifolia* [N], *A. pycnantha* [C] and *A. verticillata* [C]) and a minimum of zero soils (*A. baileyana* [N] and *A. cultriformis* [C]) **[see Supporting Information—Fig. S5 and Table S10B]**.

We found a significant interaction between soil and invasiveness category for nodulation index of effectiveness (ΔAIC  =  22.32, *w_i_*  =  1.00) **[see Supporting Information—Table S11B]**. We, therefore, examined soils individually using MCMs with Tukey contrasts and found no significant difference in nodulation index among invasiveness categories (Fig. 6B).

Visual assessment of ordination diagrams indicated no significant difference in rhizobial community composition among acacia invasiveness categories (Fig. 7B). PerManova results lent further support to this conclusion, with no significant difference in rhizobial community composition found among invasiveness categories (ADONIS, *R*  =  0.08, *P * =  0.21). In addition, we found no significant difference in rhizobial richness among categories of invasiveness (*F*  =  1.224, *P * =  0.31).

### Summary of results: native and introduced experiments

Biomass results from the native experiment showed no significant difference in aboveground biomass among acacia invasiveness categories except in one soil. Survival did not differ among categories in both the native and introduced experiments. Nodule presence and index of effectiveness was generally high across all invasiveness categories for the native experiment, but low for the introduced experiment. We found no circumstances in which multiple models were equally likely for individual response variables (i.e. differed by <2, see above) for either the native or introduced experiments. Rhizobial composition differed slightly among invasiveness categories in the native experiment only; richness did not vary among categories for either the native or introduced experiment.

## Discussion

The goal of this study was to examine whether variation in host promiscuity with rhizobial symbionts plays a role in the differential invasion of *Acacia* species in California. We found that host promiscuity as measured by plant growth in the native experiment, and survival and nodulation response in both native and introduced experiments did not differ among acacia invasiveness categories. However, acacias in the native experiment (regardless of invasive status) were able to develop nodules in a greater number of soils than in the introduced range experiment. We found limited variation in rhizobial associations among acacias that vary in invasiveness in California. While rhizobial community composition differed slightly among acacia invasiveness categories in the native experiment, rhizobial richness or the number of strains with which host species in these groups formed an association was not significantly different. Results from the introduced experiment showed no difference in community composition or richness of rhizobia associating with *Acacia* species in different invasiveness categories. Plant growth response, paired with belowground rhizobial richness results, suggests that variation in host promiscuity may not be a major determinant of invasiveness of Australian *Acacia* species in California.

Results from T-RFLP analyses indicated a slight difference in the rhizobial communities acacias in different invasiveness categories associated with, when paired with Australian soils. However, no such differences were evident with Californian soils, perhaps reflecting a greater diversity of compatible rhizobial strains in Australian soils. We found no difference in rhizobial richness among invasiveness categories for either set of experiments. Together, these results suggest that partner choice as opposed to partner breadth may be more important in explaining how interactions with rhizobia influence potential for invasiveness in this set of *Acacia* species. However, more work is required to generalize these observations. Birnbaum *et al.* (2012) found similar results when examining acacias that have become invasive within their native continent; species examined associated with the same abundance of rhizobial strains in both native and novel ranges, and for two species tested (*A. longifolia* and *A. melanoxylon*), they associated with similar rhizobial communities between ranges.

In the native range experiment, rates of nodulation and survival were similarly high across almost all soils. Although we paired acacias with soils in which they did not occur in their native range, effective rhizobial strains may be broadly distributed, as has been previously found with rhizobial (Barrett *et al.* 2012) and mycorrhizal fungal symbionts of acacias in Australia (Birnbaum *et al.* 2014). In their introduced range, acacias may be more likely to encounter rhizobial strains that are more distantly related to those with which they have co-evolved, or appropriate strains may be completely absent, such that it is more difficult to find suitable partners (perhaps partially explaining the generally lower nodulation rates we observed in the introduced experiment).

In addition to rhizobia, other organisms in soil communities may have influenced plant performance. We used whole-soil inoculation treatments, which may host multiple rhizobial symbionts as well as pathogens and other mutualistic microorganisms, and plant response may be influenced by the presence of such organisms (Thrall *et al.* 2007). The presence of non-rhizobial mutualists may have had a greater effect on plant performance in the experiment utilizing Australian soils, because acacias native to Australia likely have higher compatibility with the resident microorganisms. The presence of pathogenic organisms such as fungi or nematodes as well as interactions among co-occurring soil biota may also affect *Acacia* species growth response, influencing the potential positive benefit of being a promiscuous rhizobial host. However, whether pathogenic interactions are more likely to have influenced plant growth in Australian or Californian soils is difficult to assess. Other, more complex synergistic or antagonistic interactions may also occur when using whole soil inoculations. For example, rhizobial competition arising from the presence of multiple rhizobial genotypes within soils may have influenced mutualistic outcomes. Barrett *et al.* (2014) found evidence that acacias paired with multiple rhizobial strains suffered diminished plant growth response, likely due to altered patterns of rhizobial association. Hence, an important caveat is that we are unable to tease apart the complex species interactions that may occur among the myriad organisms occurring in natural soil communities, and which may have influenced plant performance in this study.

A previous study has shown that more invasive *Acacia* species are more promiscuous rhizobial hosts. Klock *et al.* (2015) paired 12 rhizobial strains ranging in effectiveness with 12 *Acacia* species differing in global invasiveness (four invasive, four naturalized and four casual species). In regard to plant growth, invasive acacias were generally more promiscuous hosts, able to associate and have a positive growth response with more rhizobial strains than naturalized and casual acacias. However, in this previous study, acacias were paired with single rhizobial genotypes rather than whole soil inoculations and acacia invasiveness was categorized on a global, rather than regional scale (Klock *et al.* 2015). *Acacia* species tested in this study vary in invasiveness in California; however, all except for one are invasive in at least one region of the world (Richardson and Rejmánek 2011; Rejmánek and Richardson 2013). We were interested in what drives differences in invasiveness on a regional scale; however, since all *Acacia* species tested here are invasive at least somewhere in the world, they may very well all be promiscuous rhizobial hosts, constrained by mechanisms other than host selectivity for rhizobia from becoming invasive in California. Host promiscuity with rhizobia may indeed influence the ability of acacias to invade novel regions, but other biotic and abiotic factors likely contribute to the establishment and colonization of these species, limiting some species from invading particular regions, and promoting the invasiveness of others.

The lack of aboveground biomass data in the experiment using soils collected in California reduced our ability to determine whether patterns of plant performance are consistent between native and introduced experiments. While we were able to assess the ability of acacias in different invasiveness categories to nodulate with rhizobia and their subsequent survival, we do not know whether this resulted in a beneficial growth response in the introduced range experiment. This limits our ability to assess whether acacias in the introduced range responded in a beneficial manner as a result of being paired with unfamiliar rhizobial symbionts. Future studies would benefit from assessing aboveground biomass of acacias paired with soils from their introduced range. Still, results from our nodulation, survival and molecular analyses provide strong evidence that acacias in different invasiveness categories tested here do not vary in host promiscuity with rhizobial symbionts.

Rhizobia-related mechanisms other than host promiscuity may influence the invasiveness of acacias introduced to novel regions. There is increasing evidence that some legumes have been introduced abroad with their native rhizobial symbionts (Rodríguez-Echeverría 2010; Crisóstomo *et al.* 2013; Ndlovu *et al.* 2013) and for similarity in associated rhizobial strains across native and novel ranges (Birnbaum *et al.* 2016). The introduction of both invasive species and their co-evolved beneficial symbionts may circumvent any need for introduced species to develop novel mutualistic rhizobial associations. *Acacia pycnantha*, a native Australian species that has become invasive in South Africa (Ndlovu *et al.* 2013), has been found to associate with rhizobial strains more closely related to those of Australian origin (Ndlovu *et al.* 2013). Both *A. longifolia* and *A. saligna* associate with rhizobia of Australian origin in Portugal (Rodríguez-Echeverría 2010; Crisóstomo *et al.* 2013). Legumes native to Portugal were also found to form associations with rhizobial strains of Australian origin in areas where *A. longifolia* occurred (Rodríguez-Echeverría 2010). Birnbaum *et al.* (2016) found evidence for three *Acacia* species associating with the same rhizobial strains between native and novel ranges within their native continent*.* Dual invasion of symbiotic plant and microbial species may thus be occurring in regions where acacias have been introduced, or certain rhizobial strains may be particularly widespread, potentially contributing to both above and belowground structural changes in native habitat composition.

Acacias that become invasive in California may benefit from mutualistic interactions other than the legume–rhizobia symbiosis that aid in their establishment and colonization. As indicated here, host promiscuity with rhizobia alone does not appear to delineate invasiveness of acacias in California. However, as a general trait promoting invasiveness, host interactions with other taxa may be important to the establishment, colonization and survival of these species. Ant mutualists may aid in seed dispersal and seed bank accumulation as well as protection from herbivores for *Acacia* species that become invasive in their novel range (Holmes 1990; Montesinos and Castro 2012). *Acacia* species that have become invasive in California may also develop successful mutualisms with avian seed dispersers (Glyphis *et al.* 1981; Underhill and Hofmeyr 2007; Aslan and Rejmánek 2010). Being hosts for a variety of mutualistic organisms may increase the opportunity for *Acacia* species to develop self-sustaining, spreading populations that invade novel ranges.

## Conclusions

Species that have become invasive in multiple areas of the world may be constrained from establishing and colonizing all regions where they are introduced. Identifying as well as ruling out potential mechanisms influencing expansion of species that have become invasive globally but are constrained regionally can inform management of species introduced abroad. We found that acacias varying in invasiveness in California do not differ in their ability to form symbioses with nitrogen-fixing bacteria, as evidenced by a lack of difference in plant performance and rhizobial richness when paired with diverse soil inoculants. Invasive status of introduced acacias in California, therefore, does not appear to be determined solely by the ability to associate with larger numbers of rhizobial symbionts.

Due to the demonstrated capacity of almost all *Acacia* species introduced to California to invade at least one other region of the world, and previous research showing that globally invasive acacias are promiscuous hosts, all *Acacia* species, whether currently invasive or not in California should be monitored closely for further colonization and expansion in their introduced range. Just as species differentially establish in their native ranges, the levels of invasiveness that species accomplish when introduced abroad may also vary. Our results suggest that taking scale into account when examining the factors that drive invasion of species is important; those species that are deemed invasive on a global scale may not be so on a regional scale, and different mechanisms may be influencing their capacity to invade novel regions. By identifying the mechanisms that both promote and constrain acacia invasion in particular regions, we can better inform management and future introduction of these species abroad, thereby mitigating their potential to cause negative impacts on native communities.

## Supplementary Material

Supplementary Data

## Supporting Information

The following additional information is available in the online version of this article —

**Table S1.** Soil collection sites for *Acacia* species inoculants in Australia and California.

**Table S2.** GLMM Models predicting difference in aboveground biomass (g) among California invasive rankings.

**Table S3.** Average aboveground biomass (g) and SEM of all *Acacia* species replicates in each invasiveness category when grown with each soil in the native experiment.

**Table S4.** Average aboveground biomass (g) and SEM of each *Acacia* species when grown with each soil in the native experiment.

**Table S5.** GLMM Models predicting difference in survival among California invasive rankings in the native and introduced experiments.

**Table S6.** Average percent survival (in decimal percentage) and SEM of all *Acacia* species replicates in each invasiveness category when grown with each soil in the native and introduced experiments.

**Table S7.** Average percent survival (in decimal percentage) and SEM of each *Acacia* species when grown with each soil in the native and introduced experiments.

**Table S8.** GLMM models predicting difference in nodulation presence among California invasive rankings in the native and introduced experiments.

**Table S9.** Average percent nodulation (in decimal percentage) and SEM of all *Acacia* species replicates in each invasiveness category when grown with each soil in the native and introduced experiments.

**Table S10.** Average percent nodulation (in decimal percentage) and SEM of each *Acacia* species when grown with each soil in the native and introduced experiments.

**Table S11.** GLMM models predicting difference in nodulation index among California invasive rankings in the native and introduced experiments.

**Figure S1.** Aboveground biomass (g) response of individual host species to different soil treatments.

**Figure S2.** Percent survival for each host species × soil treatment combination in the native experiment.

**Figure S3.** Percent survival for each host species × soil treatment combination in the introduced experiment.

**Figure S4.** Percent nodulation for each host species × soil treatment combination in the native experiment.

**Figure S5.** Percent nodulation for each host species × soil treatment combination in the introduced experiment.
