# Supplementary material for: Differential plant invasiveness is not always driven by host promiscuity with bacterial symbionts
Source: AoB Plants. 2016 Aug 17;8:plw060. doi: 10.1093/aobpla/plw060 (PMC5018393; doi:10.1093/aobpla/plw060)
Supplement: Supplementary Data [file supp_8_plw060_index.html]

Differential plant invasiveness is not always driven by host promiscuity with bacterial symbionts — Supplementary Data 

# Differential plant invasiveness is not always driven by host promiscuity with bacterial symbionts

## Supplementary Data

files

- Supplementary Data - zip file
