# Supplementary material for: Differential plant invasiveness is not always driven by host promiscuity with bacterial symbionts
Source: AoB Plants. 2016 Aug 17;8:plw060. doi: 10.1093/aobpla/plw060 (PMC5018393; doi:10.1093/aobpla/plw060)
Supplement: Supplementary Data [file supp_plw060_suppl_data.zip › aobplants-16068-s01.docx]

# SUPPORTING INFORMATION

**Table S1.** Soil collection sites for *Acacia* species inoculants in Australia and California.

| Code | Description | Region |
| --- | --- | --- |
| CS | Riparian | Australia |
| GE | Agricultural | Australia |
| HV | Agricultural grassland | Australia |
| ND | Diverse native habitat | Australia |
| NG | Undisturbed grassland | Australia |
| NL | *Daviesia* spp. dominated | Australia |
| PB | Abandoned pine plantation | Australia |
| SG | Eucalyptus understory | Australia |
| SN | Eucalyptus snowy field | Australia |
| YC | Eucalyptus legume understory | Australia |
| BR | Cattle pasture | California |
| CC | Native grassland/oak woodland | California |
| EP | Riparian | California |
| HD | Native shrub | California |
| OM | Willow dominated riparian | California |
| RF | Redwood forest | California |
| SP | Eucalyptus understory | California |
| TG | Native grassland | California |
| TS | Native serpentine | California |
| WH | Disturbed hillside/Broom invasion | California |

**Table S2.** GLMM models predicting difference in aboveground biomass (g) among California invasive rankings. SO = Soil, CA = California invasive ranking, SP = Species. SP is included in all models as a random effect. Model 1 tests for the presence of an interaction between SO and CA. Aikake weights (w_i_) indicate the model with the highest relative likelihood of being the best model (the closest w_i_ to 1 is the best model).

| Model number | Number of model parameters | Model variables | AIC | Delta | w_i_ |
| --- | --- | --- | --- | --- | --- |
| **1** | 3 | SO*CA; SP | 1812.0 | 0.00 | 1.00 |
| **2** | 2 | SO; SP | 1831.1 | 19.1 | 7.12 x 10^-5^ |
| **3** | 3 | SO+CA; SP | 1833.4 | 21.4 | 2.25 x 10^-5^ |
| **4** | 1 | SP | 2145.5 | 333.5 | 3.81 x 10^-73^ |
| **5** | 2 | CA; SP | 2147.4 | 335.4 | 1.48 x 10^-73^ |

**Table S3.** Average aboveground biomass (g) and SEM of all *Acacia* species replicates in each invasiveness category when grown with each soil in the native experiment.

|  | Invasive | | Naturalized | | Casual | |
| --- | --- | --- | --- | --- | --- | --- |
| Soil | Mean | SEM | Mean | SEM | Mean | SEM |
| CS | 1.17 | 0.18 | 2.67 | 0.48 | 1.07 | 0.17 |
| GE | 0.77 | 0.13 | 0.89 | 0.22 | 0.58 | 0.13 |
| HV | 0.57 | 0.13 | 0.82 | 0.23 | 0.53 | 0.11 |
| ND | 1.34 | 0.20 | 2.15 | 0.32 | 1.03 | 0.15 |
| NG | 0.33 | 0.07 | 1.07 | 0.21 | 0.31 | 0.05 |
| NL | 1.66 | 0.19 | 1.76 | 0.32 | 1.15 | 0.15 |
| PB | 1.25 | 0.19 | 1.42 | 0.22 | 0.84 | 0.12 |
| SG | 3.19 | 0.27 | 3.87 | 0.52 | 2.26 | 0.30 |
| SN | 0.74 | 0.12 | 1.21 | 0.23 | 0.82 | 0.13 |
| YC | 3.58 | 0.34 | 3.58 | 0.61 | 1.87 | 0.31 |


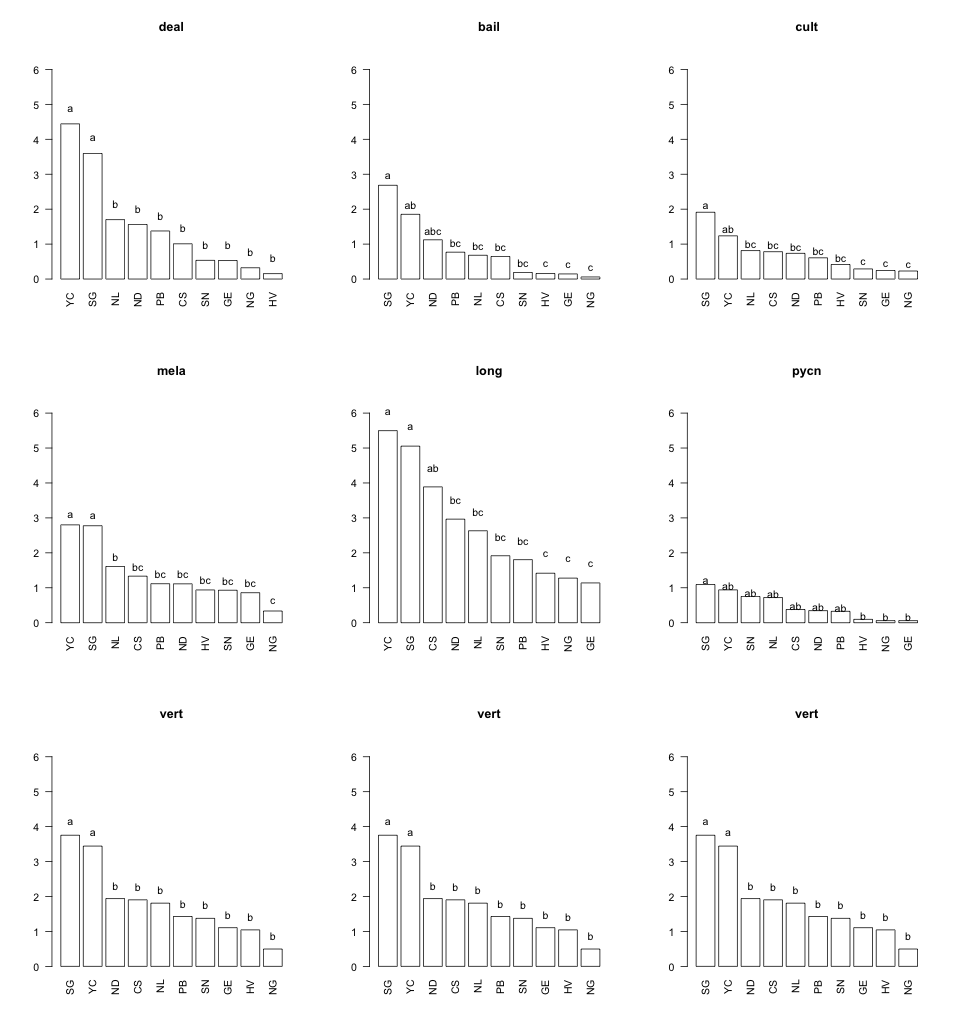


Soils

Soils

Soils

Biomass (g)

Biomass (g)

Biomass (g)

**Figure S1.** Average aboveground biomass (g) response of individual host species replicates to different soil treatments. The first column is invasive species, the second column is naturalized species, and the third column is casual species. bail = *A. baileyana*, cult = *A. cultriformis*, deal = *A. dealbata*, long = *A. longifolia*, mela = *A. melanoxylon*, pycn = *A. pycnantha*, and vert = *A. verticilata*. Letters indicate soils where *Acacia* species/replicates differ significantly in average biomass.

**Table S4.** Average aboveground biomass (g) and SEM of each *Acacia* species when grown with each soil in the native experiment.

|  | *A. baileyana* | | *A. cultriformis* | | *A. dealbata* | | *A. longifolia* | | *A. melanoxylon* | | *A. pycnantha* | | *A. verticillata* | |
| --- | --- | --- | --- | --- | --- | --- | --- | --- | --- | --- | --- | --- | --- | --- |
| Soil | Mean | SEM | Mean | SEM | Mean | SEM | Mean | SEM | Mean | SEM | Mean | SEM | Mean | SEM |
| CS | 0.65 | 0.14 | 0.78 | 0.16 | 1.01 | 0.22 | 3.89 | 0.58 | 1.33 | 0.29 | 0.38 | 0.14 | 1.91 | 0.14 |
| GE | 0.15 | 0.05 | 0.25 | 0.09 | 0.53 | 0.26 | 1.14 | 0.33 | 0.86 | 0.14 | 0.06 | 0.02 | 1.11 | 0.02 |
| HV | 0.16 | 0.09 | 0.42 | 0.11 | 0.16 | 0.04 | 1.42 | 0.36 | 0.94 | 0.17 | 0.10 | 0.04 | 1.05 | 0.04 |
| ND | 1.12 | 0.24 | 0.74 | 0.15 | 1.56 | 0.38 | 2.96 | 0.41 | 1.11 | 0.09 | 0.35 | 0.09 | 1.94 | 0.09 |
| NG | 0.06 | 0.02 | 0.23 | 0.06 | 0.32 | 0.14 | 1.28 | 0.28 | 0.34 | 0.06 | 0.06 | 0.01 | 0.50 | 0.01 |
| NL | 0.68 | 0.27 | 0.82 | 0.19 | 1.70 | 0.29 | 2.63 | 0.38 | 1.61 | 0.26 | 0.72 | 0.14 | 1.81 | 0.14 |
| PB | 0.77 | 0.16 | 0.61 | 0.16 | 1.38 | 0.36 | 1.81 | 0.32 | 1.12 | 0.13 | 0.33 | 0.08 | 1.43 | 0.08 |
| SG | 2.68 | 0.70 | 1.91 | 0.33 | 3.60 | 0.46 | 5.05 | 0.58 | 2.78 | 0.26 | 1.09 | 0.30 | 3.75 | 0.30 |
| SN | 0.19 | 0.05 | 0.29 | 0.05 | 0.53 | 0.14 | 1.92 | 0.23 | 0.93 | 0.17 | 0.75 | 0.17 | 1.37 | 0.17 |
| YC | 1.85 | 0.44 | 1.24 | 0.28 | 4.44 | 0.55 | 5.50 | 0.80 | 2.80 | 0.24 | 0.94 | 0.31 | 3.44 | 0.31 |

**Table S5.** GLMM models predicting difference in survival among California invasive rankings in the (A) native and (B) introduced experiments. SO = Soil, CA = California invasive ranking, SP = Species. SP is included in all models as a random effect. Aikake weights (w_i_) indicate the model with the highest relative likelihood of being the best model (the closest w_i_ to 1 is the best model).

A

| Model number | Number of model parameters | Model variables | AIC | Delta | w_i_ |  |
| --- | --- | --- | --- | --- | --- | --- |
| **1** | 2 | SO; SP | 543.37 | 0.00 | 0.87 |  |
| **2** | 3 | SO+CA; SP | 547.24 | 3.87 | 0.13 |  |
| **3** | 3 | SO*CA; SP | 568.37 | 25.00 | 3.26 x 10^-06^ |  |
| **4** | 1 | SP | 711.71 | 168.34 | 2.44 x 10^-37^ |  |
| **5** | 2 | CA; SP | 715.57 | 172.20 | 3.54 x 10^-38^ |  |

B

| Model number | Number of model parameters | Model variables | AIC | Delta | w_i_ |
| --- | --- | --- | --- | --- | --- |
| **1** | 2 | SO; SP | 463.08 | 0.00 | 0.79 |
| **2** | 3 | SO+CA; SP | 465.81 | 2.73 | 0.20 |
| **3** | 3 | SO*CA; SP | 475.50 | 12.42 | 0.01 x 10^-01^ |
| **4** | 1 | SP | 584.48 | 121.40 | 3.46 x 10^-27^ |
| **5** | 2 | CA; SP | 587.26 | 124.18 | 8.61 x 10^-28^ |

**Table S6.** Average percent survival (in decimal percentage) and SEM of all *Acacia* species replicates in each each invasiveness category when grown with each soil in the (A) native and (B) introduced experiments.

A

|  | Invasive | | Naturalized | | Casual | |
| --- | --- | --- | --- | --- | --- | --- |
| Soil | Mean | SEM | Mean | SEM | Mean | SEM |
| CS | 0.90 | 0.07 | 0.80 | 0.09 | 0.77 | 0.08 |
| GE | 0.65 | 0.11 | 0.55 | 0.11 | 0.62 | 0.09 |
| HV | 0.90 | 0.07 | 0.85 | 0.08 | 0.93 | 0.05 |
| ND | 1.00 | 0.00 | 0.90 | 0.07 | 0.97 | 0.03 |
| NG | 0.40 | 0.11 | 0.53 | 0.11 | 0.64 | 0.09 |
| NL | 1.00 | 0.00 | 0.80 | 0.09 | 0.90 | 0.06 |
| PB | 0.80 | 0.09 | 0.80 | 0.09 | 0.90 | 0.06 |
| SG | 1.00 | 0.00 | 1.00 | 0.00 | 0.93 | 0.05 |
| SN | 0.90 | 0.07 | 0.85 | 0.08 | 0.93 | 0.05 |
| YC | 0.95 | 0.05 | 0.95 | 0.05 | 1.00 | 0.00 |
| B |  |  |  |  |  |  |
|  | Invasive | | Naturalized | | Casual | |
| Soil | Mean | SEM | Mean | SEM | Mean | SEM |
| BR | 0.92 | 0.07 | 0.90 | 0.07 | 0.95 | 0.05 |
| CC | 0.67 | 0.13 | 0.85 | 0.08 | 0.89 | 0.07 |
| EP | 0.73 | 0.12 | 0.50 | 0.11 | 0.68 | 0.11 |
| HD | 0.93 | 0.07 | 0.70 | 0.11 | 1.00 | 0.00 |
| OM | 1.00 | 0.00 | 0.90 | 0.07 | 1.00 | 0.00 |
| RF | 0.86 | 0.09 | 0.65 | 0.11 | 0.89 | 0.07 |
| SP | 1.00 | 0.00 | 0.67 | 0.11 | 0.89 | 0.07 |
| TG | 1.00 | 0.00 | 0.85 | 0.08 | 0.95 | 0.05 |
| TS | 0.80 | 0.11 | 0.42 | 0.11 | 0.79 | 0.10 |
| WH | 0.87 | 0.09 | 0.85 | 0.08 | 1.00 | 0.00 |
| N- | 0.14 | 0.09 | 0.32 | 0.11 | 0.17 | 0.09 |


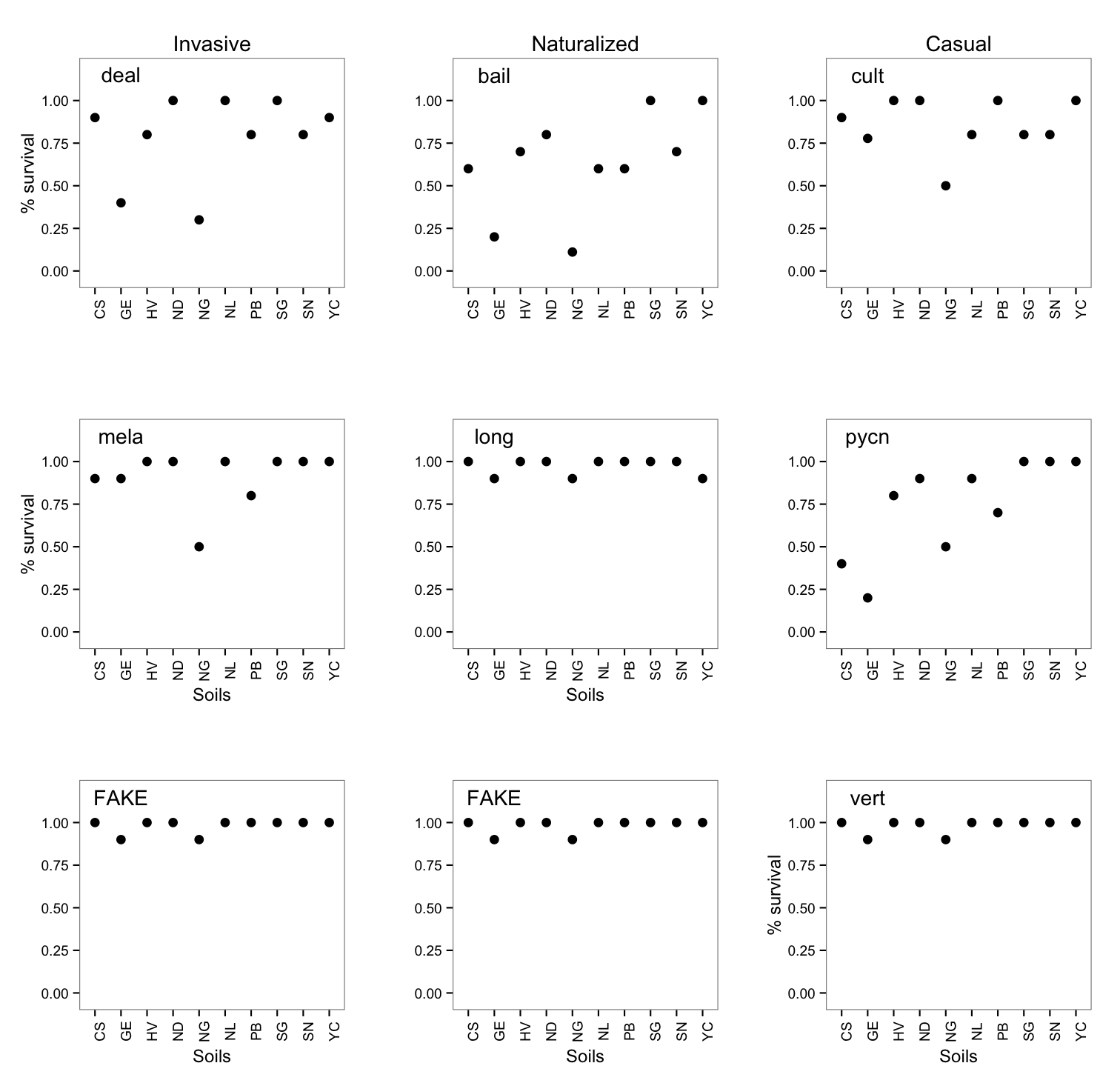


**Figure S2.** Average percent survival for each host species replicates x soil treatment combination in the native experiment. The first column is invasive species, the second column is naturalized species, and the third column is casual species. bail = *A. baileyana*, cult = *A. cultriformis*, deal = *A. dealbata*, long = *A. longifolia*, mela = *A. melanoxylon*, pycn = *A. pycnantha*, and vert = *A. verticilata*.


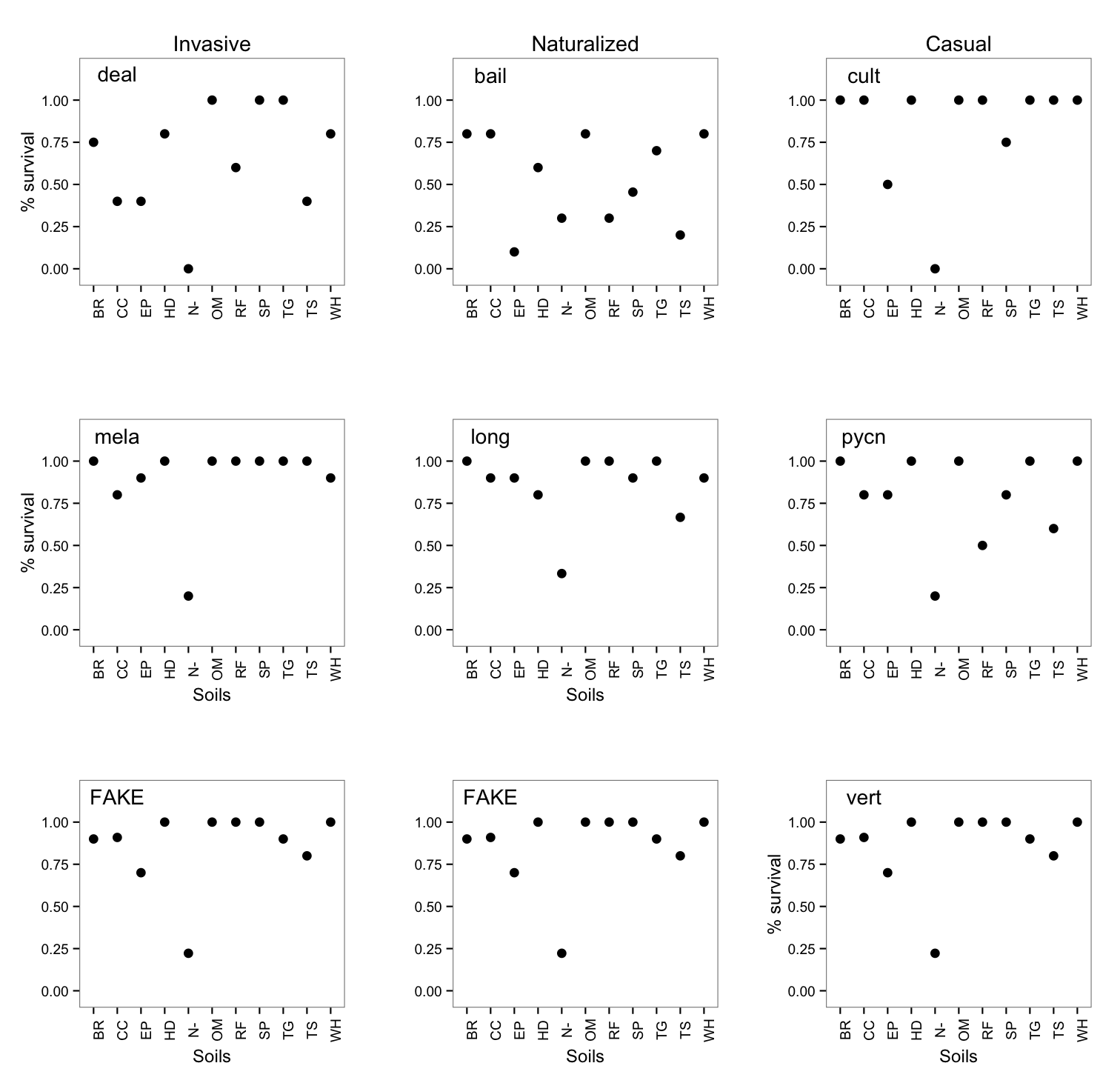


**Figure S3.** Average percent survival for each host species replicates x soil treatment combination in the introduced experiment. The first column is invasive species, the second column is naturalized species, and the third column is casual species. bail = *A. baileyana*, cult = *A. cultriformis*, deal = *A. dealbata*, long = *A. longifolia*, mela = *A. melanoxylon*, pycn = *A. pycnantha*, and vert = *A. verticilata*.

**Table S7.** Average percent survival (in decimal percentage) and SEM of each *Acacia* species when grown with each soil in the (A) native and (B) introduced experiments.

A

|  | *A. baileyana* | | *A. cultriformis* | | *A. dealbata* | | *A. longifolia* | | *A. melanoxylon* | | *A. pycnantha* | | *A. verticillata* | |
| --- | --- | --- | --- | --- | --- | --- | --- | --- | --- | --- | --- | --- | --- | --- |
| Soil | Mean | SEM | Mean | SEM | Mean | SEM | Mean | SEM | Mean | SEM | Mean | SEM | Mean | SEM |
| CS | 0.6 | 0.16 | 0.9 | 0.10 | 0.9 | 0.10 | 1.0 | 0.00 | 0.9 | 0.10 | 0.4 | 0.16 | 1.0 | 0.00 |
| GE | 0.2 | 0.13 | 0.8 | 0.14 | 0.4 | 0.16 | 0.9 | 0.10 | 0.9 | 0.10 | 0.2 | 0.13 | 0.9 | 0.10 |
| HV | 0.7 | 0.15 | 1.0 | 0.00 | 0.8 | 0.13 | 1.0 | 0.00 | 1.0 | 0.00 | 0.8 | 0.13 | 1.0 | 0.00 |
| ND | 0.8 | 0.13 | 1.0 | 0.00 | 1.0 | 0.00 | 1.0 | 0.00 | 1.0 | 0.00 | 0.9 | 0.10 | 1.0 | 0.00 |
| NG | 0.1 | 0.11 | 0.5 | 0.17 | 0.3 | 0.15 | 0.9 | 0.10 | 0.5 | 0.17 | 0.5 | 0.17 | 0.9 | 0.10 |
| NL | 0.6 | 0.16 | 0.8 | 0.13 | 1.0 | 0.00 | 1.0 | 0.00 | 1.0 | 0.00 | 0.9 | 0.10 | 1.0 | 0.00 |
| PB | 0.6 | 0.16 | 1.0 | 0.00 | 0.8 | 0.13 | 1.0 | 0.00 | 0.8 | 0.13 | 0.7 | 0.15 | 1.0 | 0.00 |
| SG | 1.0 | 0.00 | 0.8 | 0.13 | 1.0 | 0.00 | 1.0 | 0.00 | 1.0 | 0.00 | 1.0 | 0.00 | 1.0 | 0.00 |
| SN | 0.7 | 0.15 | 0.8 | 0.13 | 0.8 | 0.13 | 1.0 | 0.00 | 1.0 | 0.00 | 1.0 | 0.00 | 1.0 | 0.00 |
| YC | 1.0 | 0.00 | 1.0 | 0.00 | 0.9 | 0.10 | 0.9 | 0.10 | 1.0 | 0.00 | 1.0 | 0.00 | 1.0 | 0.00 |
| B |  |  |  |  |  |  |  |  |  |  |  |  |  |  |
|  | *A. baileyana* | | *A. cultriformis* | | *A. dealbata* | | *A. longifolia* | | *A. melanoxylon* | | *A. pycnantha* | | *A. verticillata* | |
| Soil | Mean | SEM | Mean | SEM | Mean | SEM | Mean | SEM | Mean | SEM | Mean | SEM | Mean | SEM |
| BR | 0.8 | 0.13 | 1.0 | 0.00 | 0.8 | 0.22 | 1.0 | 0.00 | 1.0 | 0.00 | 1.0 | 0.00 | 0.9 | 0.10 |
| CC | 0.8 | 0.13 | 1.0 | 0.00 | 0.4 | 0.24 | 0.9 | 0.10 | 0.8 | 0.13 | 0.8 | 0.20 | 0.9 | 0.09 |
| EP | 0.1 | 0.10 | 0.5 | 0.29 | 0.4 | 0.24 | 0.9 | 0.10 | 0.9 | 0.10 | 0.8 | 0.20 | 0.7 | 0.15 |
| HD | 0.6 | 0.16 | 1.0 | 0.00 | 0.8 | 0.20 | 0.8 | 0.13 | 1.0 | 0.00 | 1.0 | 0.00 | 1.0 | 0.00 |
| OM | 0.8 | 0.13 | 1.0 | 0.00 | 1.0 | 0.00 | 1.0 | 0.00 | 1.0 | 0.00 | 1.0 | 0.00 | 1.0 | 0.00 |
| RF | 0.3 | 0.15 | 1.0 | 0.00 | 0.6 | 0.24 | 1.0 | 0.00 | 1.0 | 0.00 | 0.5 | 0.26 | 1.0 | 0.00 |
| SP | 0.5 | 0.16 | 0.8 | 0.25 | 1.0 | 0.00 | 0.9 | 0.10 | 1.0 | 0.00 | 0.8 | 0.20 | 1.0 | 0.00 |
| TG | 0.7 | 0.15 | 1.0 | 0.00 | 1.0 | 0.00 | 1.0 | 0.00 | 1.0 | 0.00 | 1.0 | 0.00 | 0.9 | 0.10 |
| TS | 0.2 | 0.13 | 1.0 | 0.00 | 0.4 | 0.24 | 0.7 | 0.16 | 1.0 | 0.00 | 0.6 | 0.24 | 0.8 | 0.13 |
| WH | 0.8 | 0.13 | 1.0 | 0.00 | 0.8 | 0.20 | 0.9 | 0.10 | 0.9 | 0.10 | 1.0 | 0.00 | 1.0 | 0.00 |
| N- | 0.3 | 0.15 | 0.0 | 0.00 | 0.0 | 0.00 | 0.3 | 0.16 | 0.2 | 0.13 | 0.2 | 0.20 | 0.2 | 0.14 |

**Table S8.** GLMM models predicting difference in nodulation presence among California invasive rankings in the (A) native and (B) introduced experiments. SO = Soil, CA = California invasive ranking, SP = Species. SP is included in all models as a random effect. Aikake weights (w_i_) indicate the model with the highest relative likelihood of being the best model (the closest w_i_ to 1 is the best model).

A

| Model number | Number of model parameters | Model variables | AIC | Delta | w_i_ |
| --- | --- | --- | --- | --- | --- |
| **1** | 2 | SO; SP | 478.34 | 0.00 | 0.88 |
| **2** | 3 | SO+CA; SP | 482.26 | 3.92 | 0.12 |
| **3** | 3 | SO*CA; SP | 497.62 | 19.28 | 5.70 x 10^-05^ |
| **4** | 1 | SP | 576.37 | 98.03 | 4.53 x 10^-22^ |
| **5** | 2 | CA; SP | 580.31 | 101.97 | 6.31 x 10^-23^ |

B

| Model number | Number of model parameters | Model variables | AIC | Delta | w_i_ |
| --- | --- | --- | --- | --- | --- |
| **1** | 2 | SO; SP | 328.43 | 0 | 0.88 |
| **2** | 3 | SO+CA; SP | 332.42 | 3.99 | 0.12 |
| **3** | 3 | SO*CA; SP | 356.97 | 28.54 | 5.59 x 10^-07^ |
| **4** | 1 | SP | 631.81 | 303.38 | 1.17 x 10^-66^ |
| **5** | 2 | CA; SP | 635.63 | 307.2 | 1.73 x 10^-67^ |

**Table S9.** Average percent nodulation (in decimal percentage) and SEM of all *Acacia* species replicates in each each invasiveness category when grown with each soil in the (A) native and (B) introduced experiments.

A

|  | Invasive | | Naturalized | | Casual | |
| --- | --- | --- | --- | --- | --- | --- |
| Soil | Mean | SEM | Mean | SEM | Mean | SEM |
| CS | 0.95 | 0.05 | 0.80 | 0.09 | 0.83 | 0.07 |
| GE | 0.65 | 0.11 | 0.55 | 0.11 | 0.60 | 0.09 |
| HV | 0.84 | 0.08 | 0.60 | 0.11 | 0.73 | 0.08 |
| ND | 1.00 | 0.00 | 0.90 | 0.07 | 0.97 | 0.03 |
| NG | 0.45 | 0.11 | 0.50 | 0.11 | 0.63 | 0.09 |
| NL | 1.00 | 0.00 | 0.80 | 0.09 | 0.90 | 0.06 |
| PB | 0.80 | 0.09 | 0.80 | 0.09 | 0.90 | 0.06 |
| SG | 1.00 | 0.00 | 1.00 | 0.00 | 0.90 | 0.06 |
| SN | 0.90 | 0.07 | 0.79 | 0.09 | 0.93 | 0.05 |
| YC | 0.95 | 0.05 | 0.95 | 0.05 | 1.00 | 0.00 |
| B |  |  |  |  |  |  |
|  | Invasive | | Naturalized | | Casual | |
| Soil | Mean | SEM | Mean | SEM | Mean | SEM |
| BR | 0.00 | 0.00 | 0.00 | 0.00 | 0.00 | 0.00 |
| CC | 0.53 | 0.13 | 0.50 | 0.11 | 0.68 | 0.11 |
| EP | 0.27 | 0.12 | 0.45 | 0.11 | 0.63 | 0.11 |
| HD | 0.00 | 0.00 | 0.00 | 0.00 | 0.00 | 0.00 |
| OM | 0.00 | 0.00 | 0.00 | 0.00 | 0.00 | 0.00 |
| RF | 0.80 | 0.11 | 0.45 | 0.11 | 0.74 | 0.10 |
| SP | 0.43 | 0.13 | 0.50 | 0.11 | 0.67 | 0.11 |
| TG | 0.71 | 0.12 | 0.50 | 0.11 | 0.74 | 0.10 |
| TS | 0.00 | 0.00 | 0.05 | 0.05 | 0.16 | 0.09 |
| WH | 0.00 | 0.00 | 0.16 | 0.08 | 0.00 | 0.00 |
| N- | 0.00 | 0.00 | 0.00 | 0.00 | 0.00 | 0.00 |


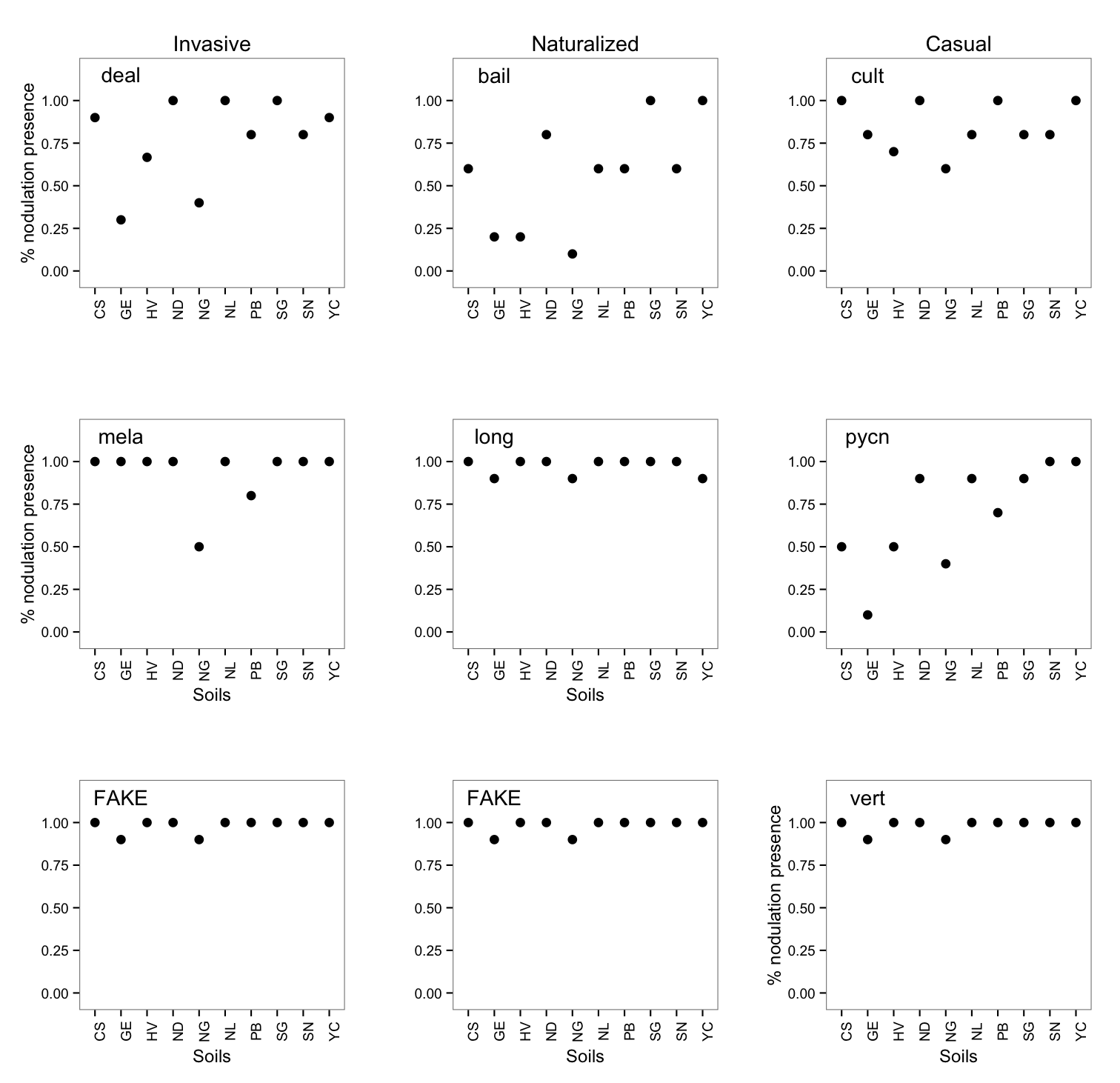


**Figure S4.** Average percent nodulation for each host species replicates x soil treatment combination in the native experiment. The first column is invasive species, the second column is naturalized species, and the third column is casual species. bail = *A. baileyana*, cult = *A. cultriformis*, deal = *A. dealbata*, long = *A. longifolia*, mela = *A. melanoxylon*, pycn = *A. pycnantha*, and vert = *A. verticilata*.


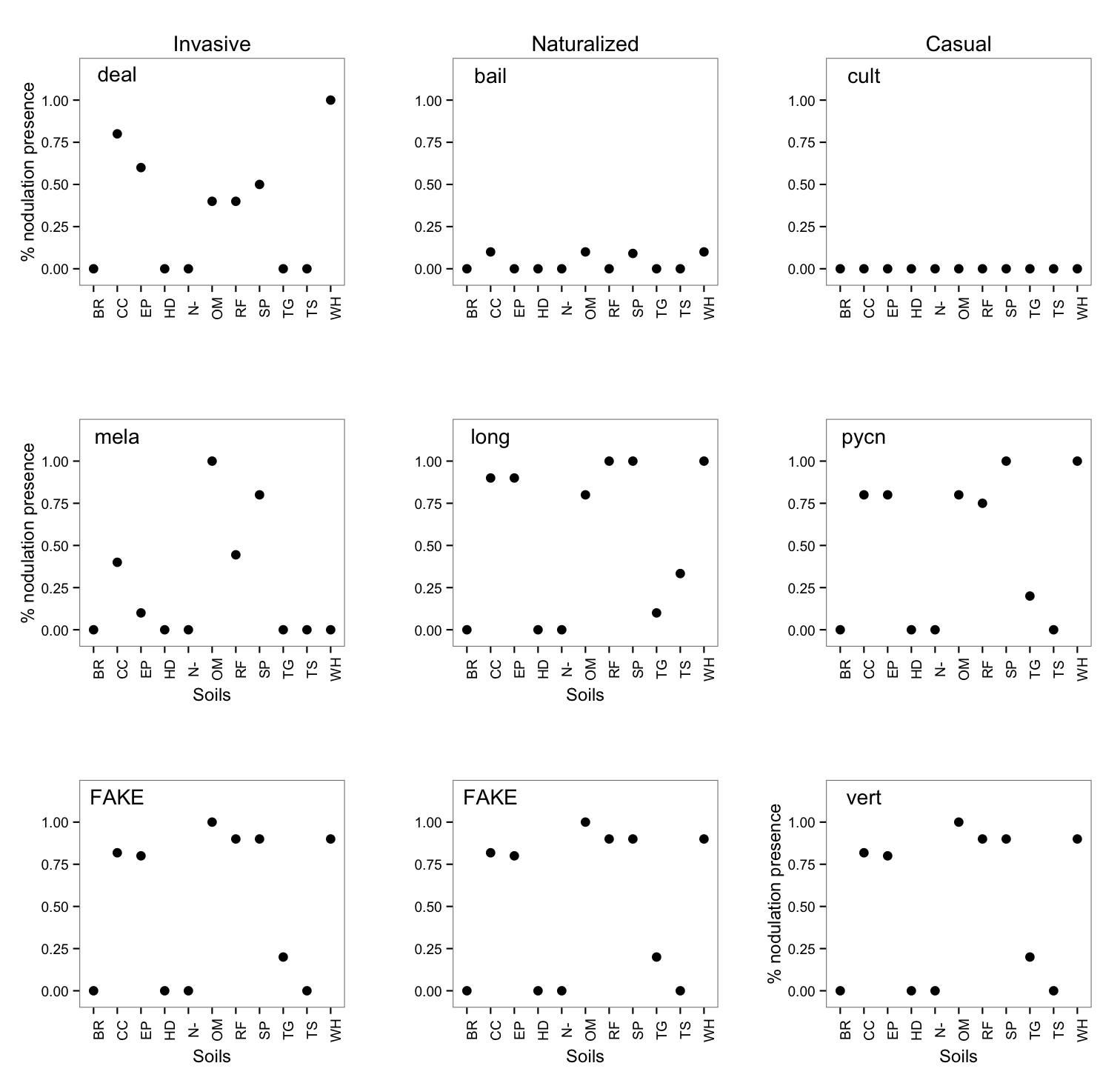


**Figure S5.** Average percent nodulation for each host species replicates x soil treatment combination in the introduced experiment. The first column is invasive species, the second column is naturalized species, and the third column is casual species. bail = *A. baileyana*, cult = *A. cultriformis*, deal = *A. dealbata*, long = *A. longifolia*, mela = *A. melanoxylon*, pycn = *A. pycnantha*, and vert = *A. verticilata*.

**Table S10.** Average percent nodulation (in decimal percentage) and SEM of each *Acacia* species when grown with each soil in the (A) native and (B) introduced experiments.

A

|  | *A. baileyana* | | *A. cultriformis* | | *A. dealbata* | | *A. longifolia* | | *A. melanoxylon* | | *A. pycnantha* | | *A. verticillata* | |
| --- | --- | --- | --- | --- | --- | --- | --- | --- | --- | --- | --- | --- | --- | --- |
| Soil | Mean | SEM | Mean | SEM | Mean | SEM | Mean | SEM | Mean | SEM | Mean | SEM | Mean | SEM |
| CS | 0.6 | 0.16 | 1.0 | 0.00 | 0.9 | 0.10 | 1.0 | 0.00 | 1.0 | 0.00 | 0.5 | 0.17 | 1.0 | 0.00 |
| GE | 0.2 | 0.13 | 0.8 | 0.13 | 0.3 | 0.15 | 0.9 | 0.10 | 1.0 | 0.00 | 0.1 | 0.10 | 0.9 | 0.10 |
| HV | 0.2 | 0.13 | 0.7 | 0.15 | 0.7 | 0.16 | 1.0 | 0.00 | 1.0 | 0.00 | 0.5 | 0.17 | 1.0 | 0.00 |
| ND | 0.8 | 0.13 | 1.0 | 0.00 | 1.0 | 0.00 | 1.0 | 0.00 | 1.0 | 0.00 | 0.9 | 0.10 | 1.0 | 0.00 |
| NG | 0.1 | 0.10 | 0.6 | 0.16 | 0.4 | 0.16 | 0.9 | 0.10 | 0.5 | 0.17 | 0.4 | 0.16 | 0.9 | 0.10 |
| NL | 0.6 | 0.16 | 0.8 | 0.13 | 1.0 | 0.00 | 1.0 | 0.00 | 1.0 | 0.00 | 0.9 | 0.10 | 1.0 | 0.00 |
| PB | 0.6 | 0.16 | 1.0 | 0.00 | 0.8 | 0.13 | 1.0 | 0.00 | 0.8 | 0.13 | 0.7 | 0.15 | 1.0 | 0.00 |
| SG | 1.0 | 0.00 | 0.8 | 0.13 | 1.0 | 0.00 | 1.0 | 0.00 | 1.0 | 0.00 | 0.9 | 0.10 | 1.0 | 0.00 |
| SN | 0.6 | 0.16 | 0.8 | 0.13 | 0.8 | 0.13 | 1.0 | 0.00 | 1.0 | 0.00 | 1.0 | 0.00 | 1.0 | 0.00 |
| YC | 1.0 | 0.00 | 1.0 | 0.00 | 0.9 | 0.10 | 0.9 | 0.10 | 1.0 | 0.00 | 1.0 | 0.00 | 1.0 | 0.00 |
| B |  |  |  |  |  |  |  |  |  |  |  |  |  |  |
|  | *A. baileyana* | | *A. cultriformis* | | *A. dealbata* | | *A. longifolia* | | *A. melanoxylon* | | *A. pycnantha* | | *A. verticillata* | |
| Soil | Mean | SEM | Mean | SEM | Mean | SEM | Mean | SEM | Mean | SEM | Mean | SEM | Mean | SEM |
| BR | 0.0 | 0.00 | 0.0 | 0.00 | 0.0 | 0.00 | 0.0 | 0.00 | 0.0 | 0.00 | 0.0 | 0.00 | 0.0 | 0.00 |
| CC | 0.1 | 0.10 | 0.0 | 0.00 | 0.8 | 0.20 | 0.9 | 0.10 | 0.4 | 0.16 | 0.8 | 0.20 | 0.8 | 0.12 |
| EP | 0.0 | 0.00 | 0.0 | 0.00 | 0.6 | 0.24 | 0.9 | 0.10 | 0.1 | 0.10 | 0.8 | 0.20 | 0.8 | 0.13 |
| HD | 0.0 | 0.00 | 0.0 | 0.00 | 0.0 | 0.00 | 0.0 | 0.00 | 0.0 | 0.00 | 0.0 | 0.00 | 0.0 | 0.00 |
| OM | 0.1 | 0.10 | 0.0 | 0.00 | 0.4 | 0.24 | 0.8 | 0.13 | 1.0 | 0.00 | 0.8 | 0.20 | 1.0 | 0.00 |
| RF | 0.0 | 0.00 | 0.0 | 0.00 | 0.4 | 0.24 | 1.0 | 0.00 | 0.4 | 0.17 | 0.8 | 0.22 | 0.9 | 0.10 |
| SP | 0.1 | 0.09 | 0.0 | 0.00 | 0.5 | 0.26 | 1.0 | 0.00 | 0.8 | 0.13 | 1.0 | 0.00 | 0.9 | 0.10 |
| TG | 0.0 | 0.00 | 0.0 | 0.00 | 0.0 | 0.00 | 0.1 | 0.10 | 0.0 | 0.00 | 0.2 | 0.20 | 0.2 | 0.13 |
| TS | 0.0 | 0.00 | 0.0 | 0.00 | 0.0 | 0.00 | 0.3 | 0.16 | 0.0 | 0.00 | 0.0 | 0.00 | 0.0 | 0.00 |
| WH | 0.1 | 0.10 | 0.0 | 0.00 | 1.0 | 0.00 | 1.0 | 0.00 | 0.0 | 0.00 | 1.0 | 0.00 | 0.9 | 0.10 |
| N- | 0.0 | 0.00 | 0.0 | 0.00 | 0.0 | 0.00 | 0.0 | 0.00 | 0.0 | 0.00 | 0.0 | 0.00 | 0.0 | 0.00 |

**Table S11.** GLMM models predicting difference in nodulation index among California invasive rankings in the (A) native and (B) introduced experiments. SO = Soil, CA = California invasive ranking, SP = Species. SP is included in all models as a random effect. Model 1 tests for the presence of an interaction between SO and CA. Aikake weights (w_i_) indicate the model with the highest relative likelihood of being the best model (the closest w_i_ to 1 is the best model).

A

| Model number | Number of model parameters | Model variables | AIC | Delta | w_i_ |
| --- | --- | --- | --- | --- | --- |
| **1** | 3 | SO*CA; SP | 1339.6 | 0.0 | 0.99 |
| **2** | 2 | SO; SP | 1349.3 | 9.7 | 0.01 |
| **3** | 3 | SO+CA; SP | 1352.2 | 12.6 | 0.01 x 10^-1^ |
| **4** | 1 | SP | 1611.7 | 272.1 | 8.13 x 10^-60^ |
| **5** | 2 | CA; SP | 1615.6 | 276.0 | 1.15 x 10^-60^ |

B

| Model number | Number of model parameters | Model variables | AIC | Delta | w_i_ |
| --- | --- | --- | --- | --- | --- |
| **1** | 3 | SO*CA; SP | 820.23 | 0 | 1.00 |
| **2** | 2 | SO; SP | 842.55 | 22.32 | 1.42 x 10^-05^ |
| **3** | 3 | SO+CA; SP | 846.3 | 26.07 | 2.18 x 10^-06^ |
| **4** | 1 | SP | 1104.55 | 284.32 | 1.82 x 10^-62^ |
| **5** | 2 | CA; SP | 1108.31 | 288.08 | 2.78 x 10^-63^ |
