# Supplementary material for: Differential plant invasiveness is not always driven by host promiscuity with bacterial symbionts
Source: AoB Plants. 2016 Aug 17;8:plw060. doi: 10.1093/aobpla/plw060 (PMC5018393; doi:10.1093/aobpla/plw060)
Supplement: Supplementary Data [file supp_plw060_suppl_data.zip › aobplants-16068-s02.docx]

**Differential plant invasiveness is not always driven by host promiscuity with bacterial symbionts**

Metha M. Klock, Luke G. Barrett, Peter H. Thrall, and Kyle E. Harms

1. **Topics selected from subject list:**

Biological Invasions

Conservation Biology

Ecology

Plant-Microbe Interactions

1. **Promotional statement:** Acacias have been widely introduced outside their native range, with a subset of species becoming invasive in multiple parts of the world. Our study examined whether a key mechanism in acacia life history, the legume-rhizobia symbiosis, influences invasiveness of these species. We determined whether more invasive acacias formed symbioses with a wider diversity of rhizobial strains (*i.e.* are more promiscuous hosts) and found that acacias introduced to California are promiscuous hosts regardless of invasive status. Our results highlight the importance of examining mechanisms driving species invasions on different scales and in their native and introduced ranges.
2. **Image:**

**
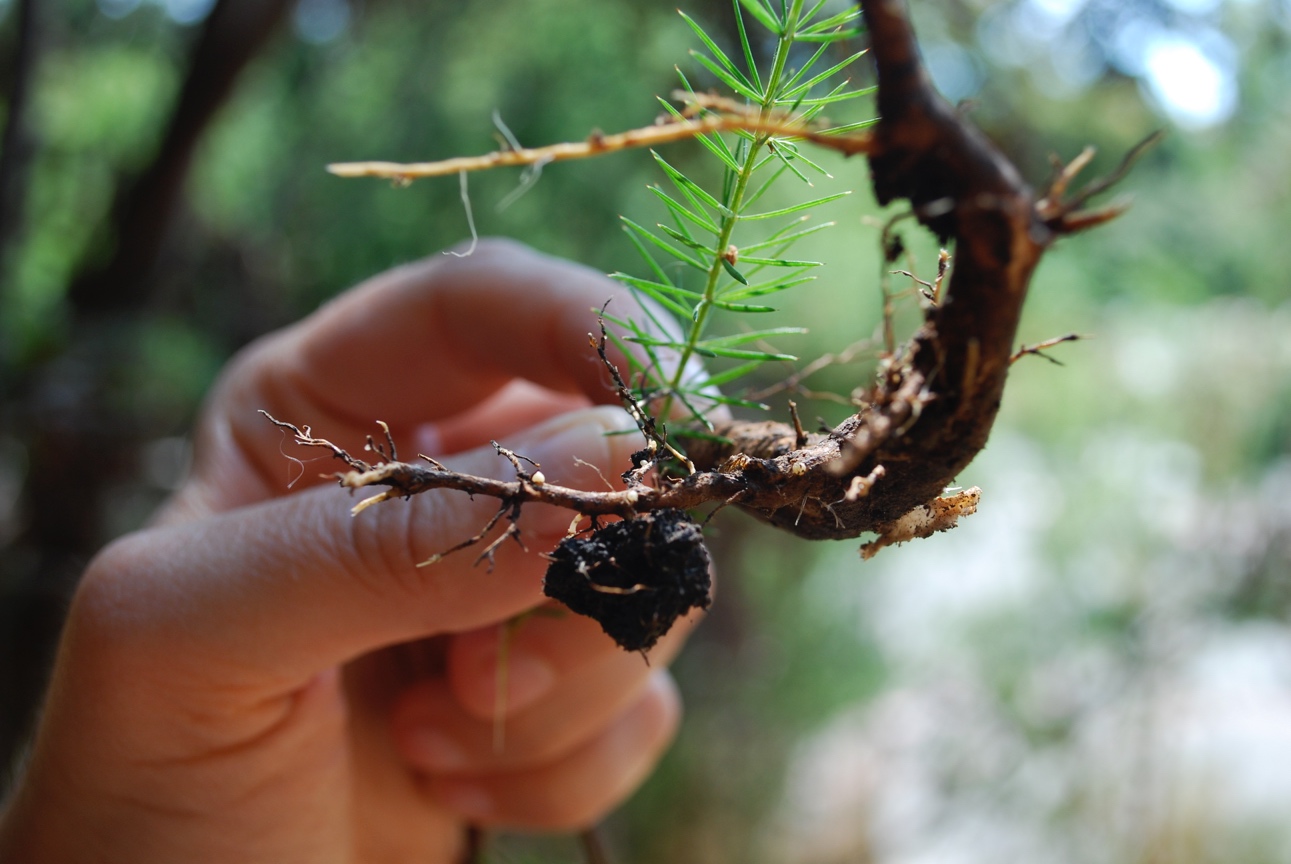
**

*Acacia verticillata* roots with small nodules (Image by Metha Klock)

1. **Email addresses of colleagues to receive a PDF:**

elizabethwandrag@gmail.com,

chbirnbaum@gmail.com,

jleroux@sun.ac.za,

mritter@calpoly.edu,

heidihirsch71@googmail.com,

joe@acaciamulga.net,

jhkeet@hotmail.com,

hollyvuong168@gmail.com

1. **Special Issue for which your paper is being prepared:**

[Evolutionary dynamics of tree invasions](http://www.oxfordjournals.org/our_journals/aobpla/Evolutionary_Dynamics_of_Tree_In.html)
