# Supplementary material for: Differential plant invasiveness is not always driven by host promiscuity with bacterial symbionts
Source: AoB Plants. 2016 Aug 17;8:plw060. doi: 10.1093/aobpla/plw060 (PMC5018393; doi:10.1093/aobpla/plw060)
Supplement: Supplementary Data [file supp_plw060_suppl_data.zip › aobplants-16068-s03.doc]

# OPEN ACCESS – RESEARCH ARTICLE

# Differential plant invasiveness is not always driven by host promiscuity with bacterial symbionts

### Metha M. Klock^1^*, Luke G. Barrett^2^, Peter H. Thrall ^2^ and Kyle E. Harms^1^

^1^Department of Biological Sciences, Louisiana State University, Baton Rouge, LA, 70803.

^2^CSIRO Agriculture Flagship, Canberra, ACT 2601, Australia.

*Corresponding author

Corresponding author’s e-mail address: klockmm@gmail.com

Running head: Differential invasiveness is not always driven by host promiscuity
